# Profiling Metabolites with Antifungal Activities from Endophytic Plant-Beneficial Strains of *Pseudomonas chlororaphis* Isolated from *Chamaecytisus albus* (Hack.) Rothm.

**DOI:** 10.3390/molecules29184370

**Published:** 2024-09-14

**Authors:** Wojciech Sokołowski, Monika Marek-Kozaczuk, Piotr Sosnowski, Ewa Sajnaga, Monika Elżbieta Jach, Magdalena Anna Karaś

**Affiliations:** 1 Department of Genetics and Microbiology, Institute of Biological Science, Faculty of Biology and Biotechnology, Maria Curie-Skłodowska University, Akademicka 19 Str., 20-033 Lublin, Poland; wojciech.v.sokolowski@gmail.com (W.S.); monika.marek-kozaczuk@mail.umcs.pl (M.M.-K.); 2 Department of Bioanalytics, Medical University of Lublin, Jaczewskiego 8b Str., 20-059 Lublin, Poland; piotr.sosnowski.phd@gmail.com; 3 Department of Biomedicine and Environmental Research, John Paul II Catholic University of Lublin, Konstantynów 1J Str., 20-708 Lublin, Poland; esajnaga@kul.pl; 4 Department of Molecular Biology, John Paul II Catholic University of Lublin, Konstantynów 1I Str., 20-708 Lublin, Poland; monika.jach@kul.pl

**Keywords:** *Pseudomonas chlororaphis*, fungal biocontrol, phenazines, pyrrolnitrin, VOCs, LC-MS/MS

## Abstract

Fungal phytopathogens represent a large and economically significant challenge to food production worldwide. Thus, the application of biocontrol agents can be an alternative. In the present study, we carried out biological, metabolomic, and genetic analyses of three endophytic isolates from nodules of *Chamaecytisus albus*, classified as *Pseudomonas chlororaphis* acting as antifungal agents. The efficiency of production of their diffusible and volatile antifungal compounds (VOCs) was verified in antagonistic assays with the use of soil-borne phytopathogens: *B. cinerea*, *F. oxysporum*, and *S. sclerotiorum*. Diffusible metabolites were identified using chromatographic and spectrometric analyses (HPTLC, GC-MS, and LC-MS/MS). The *phzF*, *phzO*, and *prnC* genes in the genomes of bacterial strains were confirmed by PCR. In turn, the plant growth promotion (PGP) properties (production of HCN, auxins, siderophores, and hydrolytic enzymes, phosphate solubilization) of pseudomonads were bioassayed. The data analysis showed that all tested strains have broad-range antifungal activity with varying degrees of antagonism. The most abundant bioactive compounds were phenazine derivatives: phenazine-1-carboxylic acid (PCA), 2-hydroxy-phenazine, and diketopiperazine derivatives as well as *ortho*-dialkyl-aromatic acids, pyrrolnitrin, siderophores, and HCN. The results indicate that the tested *P. chlororaphis* isolates exhibit characteristics of biocontrol organisms; therefore, they have potential to be used in sustainable agriculture and as commercial postharvest fungicides to be used in fruits and vegetables.

## 1. Introduction

Soil-borne fungal pathogens cause enormous wastage in food commodities or crop losses every year mainly due to readily dispersed spores [1]. In parallel, agrosystems with a strictly homogeneous genetic base, i.e., a reservoir of essentially identical plant material, make such species more susceptible to widespread devastation by fungi. As a result, harvest efficiency decreases by 10% to 23% every season, and this value is even higher when adding postharvest values. Therefore, global food security is increasingly being threatened due to the constantly expanding human population. Also, fungal infections of crops can lead to mycotoxin poisoning of consumers [2,3,4].

*Fusarium oxysporum*, *Botrytis cinerea*, and *Sclerotinia sclerotiorum* are among the most devastating causative agents of several plant diseases highly reducing farmers’ profits. *F. oxysporum* belongs to toxigenic hemibiotrophic phytopathogens developing vascular wilt on more than 150 different crop species [5,6]. Its representatives form a widely distributed and phylogenetically diverse group capable of mycotoxin production, which is ranked fifth out of the ten most lethal plant pathogens [7]. In turn, *S. sclerotiorum* and *B. cinerea* are closely related aggressive necrotrophic fungi and infection agents of white and gray mold, respectively, with broad host ranges and environmental persistence [8,9]. *B. cinerea* affects more than 1400 and *S. sclerotiorum* over 400 species, mainly dicotyledonous plants, but they can also attack monocotyledonous ones, thus becoming the main threats to agriculture systems [10,11,12,13].

Traditionally, chemical pesticides are used for the protection against or eradication of fungi. However, their effectiveness may be limited by washdown due to rain or irrigation. When they reach the soil, they have significant impacts throughout the ecosystem due to their accumulation as toxic residues for years or decades after they are applied. Another problem is that their overuse and single-site use can enhance the development of specific resistance in fungi [12]. Furthermore, due to their accumulation on or in crop plants, they can affect public health [14]. In turn, microorganism-based biological control offers an effective alternative to synthetic chemical treatment of fungal diseases. These microbial biopesticides with antagonistic activities, formulated based on biosafety microorganisms, are usually inherently less toxic than conventional pesticides and generally affect only the target pest and closely related organisms. In contrast, broad-spectrum conventional pesticides may influence organisms as different as birds, insects, and mammals. Biopesticides, which are bacterial secondary metabolites, are often effective in very small quantities and can quickly decompose resulting in lower exposures and avoidance of pollution problems caused by conventional pesticides. Additionally, because these metabolites have different modes of action, the development of fungal resistance can be reduced [12].

It is well recognized that endophytic bacteria that colonize the interior of plants can play multifaceted roles by helping in nutrient acquisition and acting specifically as fungal biocontrol agents. The special characteristics of plant growth promotion (PGP) are attributed to non-rhizobial nodule endophytes isolated from some legumes. Among these features, indirect PGP properties in the form of antagonistic effects are also mentioned, which was discussed exhaustively by Hnini et al. [15]. The most prevalent nodule endophytes belong to the genera *Pseudomonas* and *Bacillus* succeeded by *Enterobacter*, *Acinetobacter*, *Ochrobactrum*, *Paenibacillus*, *Pantoea*, *Agrobacterium*, and *Microbacterium* [15,16]. In this group, pseudomonas are ubiquitous bacteria in agricultural soils possessing numerous attributes suitable for use as biopesticides due to secretion of a wide spectrum of bioactive molecules, such as antibiotics, lytic enzymes, biosurfactants, siderophores, or volatile compounds. They can also support plants in their self-control systems by production of metabolites that trigger induced systemic defense (ISR) [17,18,19]. In particular, several strains were proposed as biofertilizer and biocontrol agents for sustainable agriculture, i.e., *Pseudomonas fluorescens*, *Pseudomonas putida*, *Pseudomonas aeruginosa*, *Pseudomonas syringae*, or *Pseudomonas chlororaphis,* thanks to production of, e.g., oomycin A, pyrrolnitrin, pyoluteorin, and 2,4-diacetylphloroglucinol. Due to the essentially nonpathogenic nature and the ability to synthesize different types of phenazines, which can suppress the growth of fungi, environmentally friendly strains such as *P. chlororaphis* and *P. fluorescens* can be the best choice [17,20]. The ease of maintenance and rapid in vitro growth of these strains, combined with their ability to quickly consume seed and root exudates, and their ability to colonize both the rhizosphere and the interior of plants by competing aggressively with other microorganisms make them suitable as biocontrol and PGP agents.

The current study is part of the project aimed at the protection of *Chamaecytisus albus* (Hacq.) Rothm. White Spanish broom is an extremely rare species in Europe; in Poland, it occurs only in two locations. These localities are found to be the farthest in the north and the west in Europe, and they are completely detached from the continuous range. Due to the decreasing number of its already small population, the plant is included in many European red lists and red books as an endangered or potentially endangered species. In Poland, the best conditions for its growth are provided by calcareous, nitrogen-poor, intensely insolated habitats of xerothermic grasslands [21]. One of the trends in research consisted of the isolation of non-rhizobial strains with biocontrol activity from nodules of *Ch. albus*, followed by metabolomic characterization of their potentially active compounds. As a result, three endophytic isolates marked as 16A1, 16B1, and 23aP were obtained, identified, and classified into the *P. chlororaphis* species, exhibiting antagonistic properties against *B. cinerea*, *F. oxysporum*, and *S. sclerotiorum*. Their biocontrol activity was confirmed using metabolomic and molecular analyses. To our knowledge, this is the first report of *P. chlororaphis* nodule strains associated with White Spanish broom. Understanding their antifungal potential can on the one hand help in the protection of the species and, on the other hand, may have a practical biotechnological aspect in the future.

## 2. Results

### 2.1. Phylogenetic Analysis and PGP Properties of Strains

The study involved endophytic strains isolated from nodules of Ch. albus. Among all the Gram-negative rod-shaped isolates, three (16A1, 16B1, 23aP) capable of forming colonies with orange pigmentation on 79CA agar were chosen for further studies. This selection was made after reviewing the current state of the art, indicating that representatives of this group of bacteria may have biocontrol potential and PGP properties [17]. Among the strains, 23aP was already identified, and on the basis of a multilocus sequence analysis (MLSA) of the combined partial sequences of three housekeeping genes (recA, gyrB, rpoB), was classified to the species *P. chlororaphis* [22]. Comparative analysis of the sequences of genes recA and gyrB of strains 16A1 and 16B1 (PQ095915, PQ095916, PQ095917, PQ095918) showed their greatest similarity (100%) to the corresponding genes of strain 23aP and indicated that all the strains studied belong to the species *P. chlororaphis*. The phylogenetic tree for strain 23aP, which is identical for new isolates, was published in our previous publication [22].

Since the phylogenetic analysis confirmed our preliminary suspicion that the tested isolates belong to a group of bacteria used in agriculture as biofertilizers and biopesticides, they were subjected to further tests to identify their plant growth promotion characteristics. In plate bioassays, all the strains showed the ability to solubilize phosphate and produce extracellular lytic enzymes (cellulases, proteases). Auxin-type indole-3-acetic acid (IAA) was produced only by strains 16B1 and 23aP (Table 1). The biosynthesis of other PGP factors, such as siderophores and hydrogen cyanide, is discussed in further sections.

### 2.2. In Vitro Detection of Antifungal Activities of Endophytes in the Antagonism Assay

**Figure 1:**
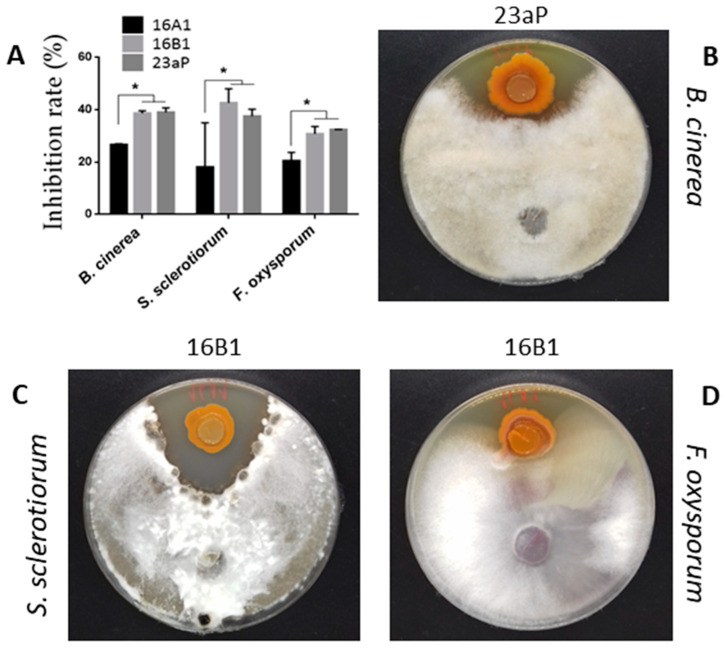
Antifungal activity of nodule endophytic *Pseudomonas* strains. (**A**) Mean inhibition of mycelial growth (%) in relation to control non-treated phytopathogen cultures. In (**A**), values are shown as mean ± SD from three independent experiments with a 95% confidence level. * means *p* < 0.05. (**B**–**D**) Representative plates for individual fungi with the largest fungistasis zones in the antagonism assays.

The results of the antagonism assay against *B. cinerea*, *F. oxysporum*, and *S. sclerotiorum* showed different-sized fungistasis zones around endophytic bacteria (Figure 1). The exact inspection of the data obtained revealed that 16B1 and 23aP had a similar range of action in relation to all the phytopathogens tested (*p* > 0.05), while 16A1 showed much lower activity (*p* < 0.05) (Figure 1A). Despite the slight differences between the strains, all exhibited broad-spectrum in vitro antifungal activities that manifested themselves in inhibition of mycelial growth. The most sensitive to the action of bacterial isolates was *S. sclerotiorum* 10Se01. The growth of *B. cinerea* 10Bc01 was inhibited in an almost similar range (*p* > 0.05), and *F. oxysporum* 10Fo01 appeared to be the most resistant.

### 2.3. Analysis of Diffusible Metabolites

The data obtained in the antagonism assay suggested that the bacterial isolates were capable of producing diffusible antifungal compounds. Therefore, cell-free ethyl acetate extracts were prepared for chromatographic and spectrometric analyses. HPTLC chromatography detected the same six band patterns for all strains with retardation factors (R_f_) of 0.66, 0.61, 0.50, 0.24, 0.21, and 0.14. However, for isolates 16B1 and 23aP, the intensity of spots with R_f_ 0.21 and 0.14 was much higher than for 16A1 (Figure S1). To make preliminary identification of the extracellular metabolites liberated by *Pseudomonas* strains, GC-MS analysis was performed. In the registered chromatograms, the peaks with the highest intensities at retention times (R_t_) 19.12 and 23.41 min represented the phenazine derivatives 2-hydroxyphenazine (2-OH-Phz) and phenazine-1-carboxylic acid (PCA), respectively (Figure S2A). Annotation was performed based on registered m/z values and matching mass fragmentation patterns with the mass spectra in the NIST (National Institute of Standards and Technology) library and in the literature [23].

Due to the limitations of the GC-MS analysis of compounds showing high boiling points and, therefore, low volatility in the conditions applied, making it impossible to separate them, the samples were additionally subjected to LC-MS/MS in the positive ion mode. The registered liquid chromatograms showed similarities in intensities of the main peaks for both 16B1 and 23aP, while in the case of 16A1, they differed in two peaks at R_t_ 5.47 and 6.76 min (Figure S2B). The identification of the compounds by monoisotopic mass analysis via alignment with database information accompanied by inspection of fragmentation patterns in LC-MS/MS revealed the presence of fifteen metabolites [24,25]. Details are included in Table 2 and Supplementary Materials (Figure S3). According to the data obtained, it appeared that the most abundant phenazine derivatives were 2-OH-Phz, PCA, and 2-hydroxy-phenazine-1-carboxylic acid (2-OH-PCA); however, phenazine (Phz), 2,8-dihydroxyphenazine (2,8-OH-Phz), phenazine-1,6-dicarboxylic acid (Phz-1,6-di-COOH), and 6-methylphenazine-1-carboxylic acid (6-methyl-PCA) were recognized as well. In the case of strains 16B1 and 23aP, the relative content of 2-OH-Phz was nearly twice higher than in strain 16A1. In turn, the relative content of PCA in 16A1 was twice as high as in the other two strains. These and the literature data [26] indicated that the spots revealed by HPTLC analysis with R_f_ 0.21 and 0.61 could represent 2-OH-Phz and PCA, respectively.

The detailed LC-MS/MS analysis of the metabolites also identified ortho-dialkyl-aromatic acids (lahorenoic acids A and C), diketopiperazine derivatives (cyclo-L-Pro-L-Val, cyclo-L-Pro-L-Met-diketopiperazine, and cyclo-L-Pro-L-Tyr syn. maculosine), 2-acetamidophenol, the quorum-sensing signal molecule (hexahydroquinoxaline-1,4-dioxide), and a trace amount of pyrrolnitrin (Table 2).

**Figure 2:**
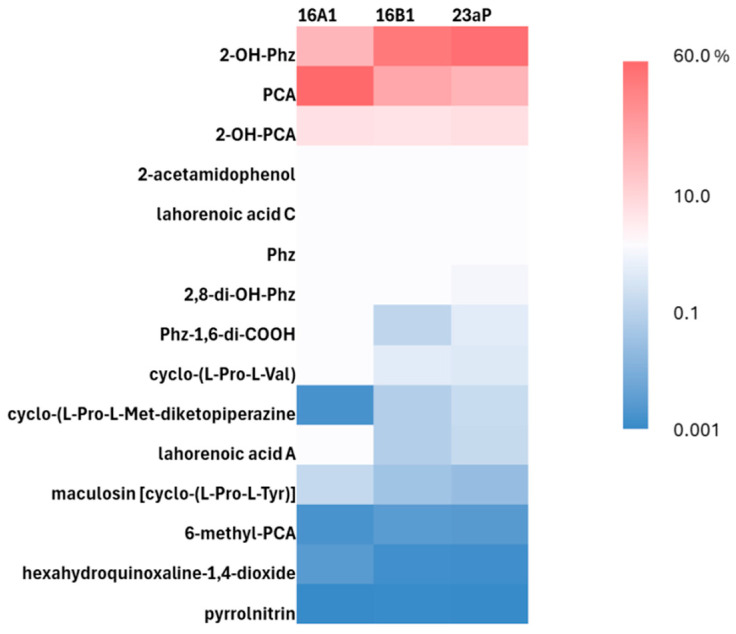
Heat map showing the relative abundance (%) of secondary metabolites identified in extracts from cell-free supernatants of 16A, 16B1, and 23aP cultures with LC-MS/MS. The color code ranging from blue to red indicates low to high relative content. Explanations of the abbreviations can be found in the text.

The above findings indicate that, in the tested culture conditions for particular isolates, there were no qualitative but only minor quantitative differences between the metabolites produced, which may result from the different growth kinetics of the strains and the regulation mechanisms of gene expression (Figure 2 and Table S1). Moreover, the almost identical chromatographic profiles of the extracts of both 16B1 and 23aP strains partly explained their similar biocontrol activity against the tested fungal phytopathogens in the antagonism assays. In turn, the weaker effect of 16A1 on the fungi could be related to the lower production of some compounds with antifungal potential in the conditions studied.

### 2.4. Molecular Characteristics of Genes for Antifungal Antibiotics

Among the 12 genes tested engaged in the biosynthesis of 10 antifungal compounds (Table S2), specific PCR products were obtained only for 3 of them, i.e., prnC a key gene in the biosynthesis of pyrrolnitrin, which has strong antifungal activity, and genes phzF and phzO involved in the synthesis of phenazine derivatives. The phylogenetic tree of 764 bp phzF gene fragments of strains 16A1, 16B1, and 23aP and twenty closely related bacteria is presented in Figure S4. The data analysis revealed that the studied strains are located on the same branch with 100.0% similarity of the phzF gene sequences. These bacteria grouped with the most closely related *P. chlororaphis* subsp. aurantiaca DSM19603^T^ (96.9% sequence similarity) and *P. chlororaphis* subsp. aureofaciens DSM6698^T^ (96.6% sequence similarity), which was supported by a high bootstrap value. The phylogenetic relationship shown by a comparative analysis of 701 bp phzO gene sequences between the bacteria studied and six closely related strains is presented in Figure S4A. The similarity of these sequences between the tested strains ranged from 99.8 to 100.0%. The tested bacteria grouped in the common branch with the most closely related *P. chlororaphis* subsp. aureofaciens DSM6698^T^ (99.4–99.5% sequence similarity). The phylogenetic analysis of 694 bp prnC gene fragments (Figure S4B) of strains 16A1, 16B1, and 23aP and 9 closely related taxa indicated from 99.8 to 100.0% similarity of gene sequences between the bacteria studied. All the tested bacteria grouped on the same branch with closely related *P. chlororaphis* subsp. aureofaciens DSM6698^T^ and *P. chlororaphis* subsp. aurantiaca DSM19603^T^ (97.9–98.1% sequence similarity) and *P. chlororaphis* subsp. aurantiaca Q16 (97.7–97.8% sequence similarity), which was supported by a high bootstrap value.

### 2.5. Siderophore Production

**Figure 3:**
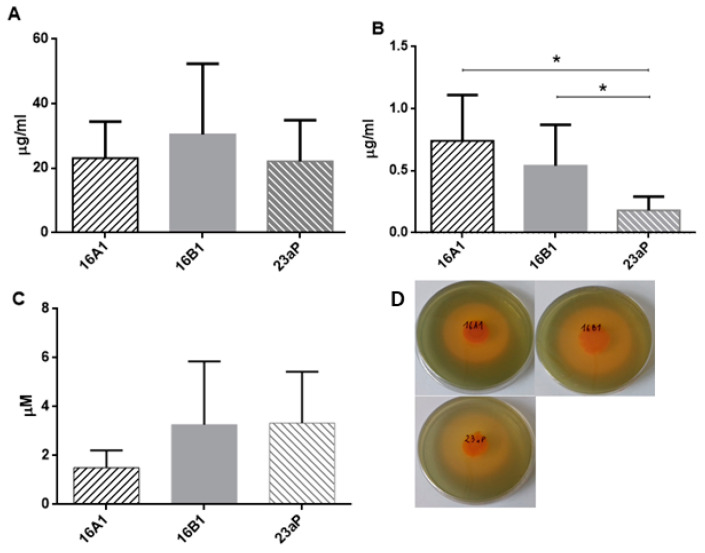
Production of siderophores by *Pseudomonas* strains: (**A**) catechol-type compared to the standard SA (*p* > 0.05, ns—not significant), (**B**) catechol-type in relation to 2,3-DHBA (*; *p* < 0.05), (**C**) pyoverdine (*p* > 0.05, ns), and (**D**) CAS assay after four days of incubation (*p* > 0.05, ns). Student’s *t*-test with a 95% confidence level was applied for statistical analysis.

The most advanced biocontrol mechanism of pathogens is suppression of their growth by secreting siderophores that efficiently sequester iron and deprive the pathogen of this element [19]. They are also known to contribute to the promotion of host plant growth and are proposed as promising sources for iron biofertilization in agriculture [27]. Therefore, strain cultures on CAS agar were performed to detect siderophore production (Figure 3D). The general rule is that the CAS medium changes color from blue to purple under the influence of catechol Fe chelators and to yellow–orange after reaction with hydroxamic ones [28]. In this study, the inspection of indicative orange halos in the CAS cultures showed that all strains were able to produce mainly hydroxamic, while the analysis of the siderophore production index (SPI) suggested a similar level of their production (*p* > 0.05) (Figure 3D). However, due to the large number of such molecules that can be secreted by the same *Pseudomonas* strain [19] and the complexity of culture supernatants, it is not possible to accurately determine the real nature and content of siderophores using the CAS assay. Therefore, their concentrations were estimated in cell-free supernatants from cultures of endophytes run in iron-limiting conditions. The production of catecholate types was calculated in relation to salicylic acid (SA) (Figure 3A) and 2,3-dihydobenzoic acid (2,3-DHBA) (Figure 3B), while the content of hydroxamate-types was measured in relation to pyoverdine (Figure 3C). The data analysis revealed that strains 16A1 and 16B1 were the best producers of chelators with the catechole group (2,3-DHBA) (Figure 3B, *p* < 0.05), and the amount of pyoverdine production was the same in all the tested strains (Figure 3C; *p* > 0.05).

### 2.6. Volatile Organic Compound (VOC) Assays

**Figure 4:**
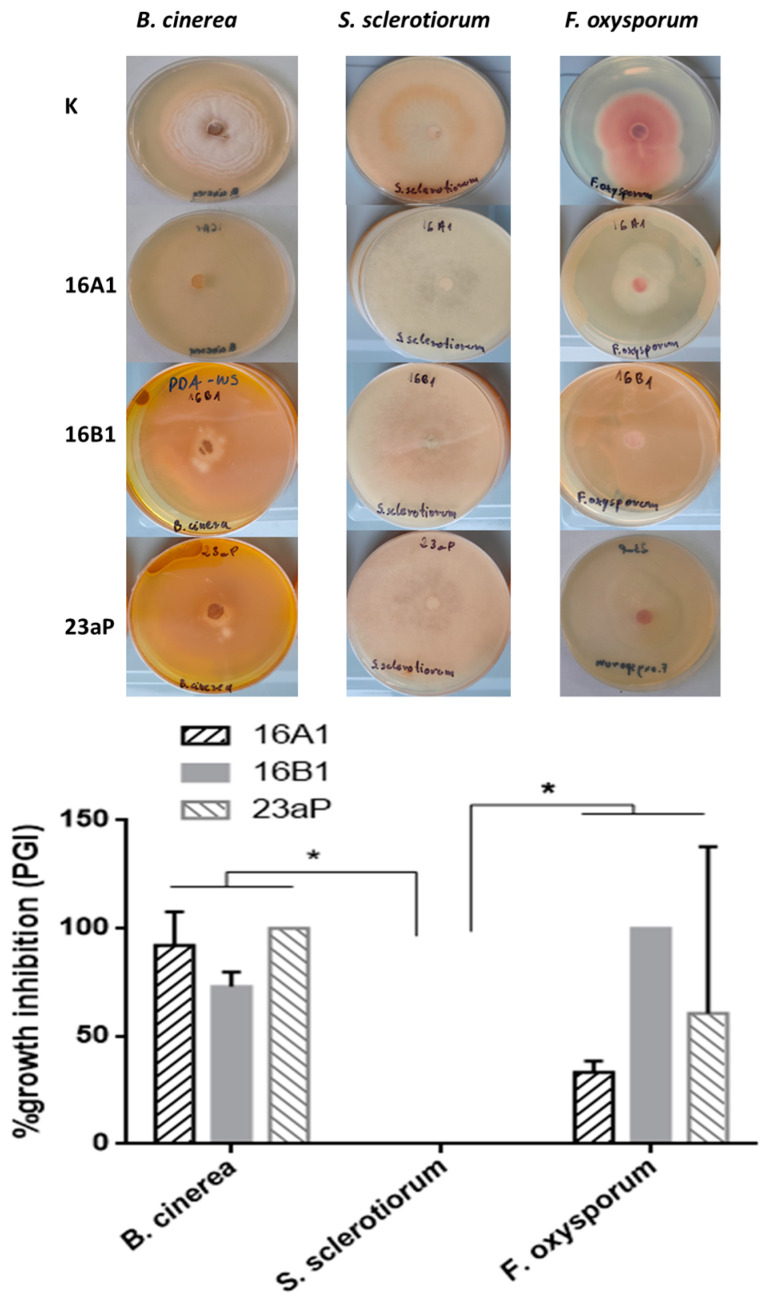
Fungistatic effects of VOCs produced by endophytic strains of *Pseudomonas*. The activity of isolated 16A1, 16B1, and 23aP was tested against three fungal strains: *B. cinerea*, *S. sclerotiorum*, and *F. oxysporum*. K—control dual-plates with fungi non-inoculated with bacteria. The error bars indicate the standard error of the mean (SEM) with a 95% confidence level, * means *p* < 0.05.

Since bacterial VOCs show biocontrol potential to inhibit mycelial growth [29], the overlapping plates were conducted. The evaluation of the data showed the most pronounced PGI range observed for *B. cinerea* (73–100%), regardless of the tested bacterial strain and significantly higher 33–100% variations in PGI in the case of *F. oxysporum*, while *S. sclerotiorum* was completely resistant to the bacterial VOCs (Figure 4; *p* < 0.05 one-way ANOVA). Because hydrogen cyanide (HCN) is one of the VOCs with very high effectiveness in the control of fungal infections by restraining the fungal cytochrome-C terminal oxidase, we took this factor into account in our studies. As a result, we established that all the strains were capable of the biosynthesis thereof (Table S1). Altogether, no differences in the fungal biocontrol effectiveness by the VOCs of particular pseudomonas strains were demonstrated (*p* > 0.05 one-way ANOVA).

## 3. Discussion

Globally, fungal infections cause plant crop diseases and postharvest decay, resulting in a substantial decrease in crop yields and quality loss [30]. Combating the causes and effects is mainly based on conventional chemical methods, which generates additional costs of production while negatively affecting the environment and reducing the health-promoting values of plant products. Recently, the use of biopesticides has gained more attention, which is driven by the need to seek for sustainable and eco-friendly alternatives to confront phytopathogens [31]. Such products based on microorganisms, especially these containing bacteria classified to the *Pseudomonas* genus, offer a more holistic approach for fungal pathogen control. The increase in registrations for biopesticides reflects the rising demand for such solutions [30,32].

Many *Pseudomonas* strains demonstrate a strong antagonism against fungal phytopathogens in parallel with plant growth promotion. In contrast to chemicals, direct toxicity is not the main mode of their action, but rather a variety of mechanisms are involved, including competition, induction of systemic resistance, and the activity of antibiotics such as phenazines, pyrrolnitrin, pyoluteorin, and 2,4-diacetyl-phloroglucinol (2,4-DAPG), to mention only the most important ones [17,33]. In the present study, we were unable to obtain gene amplicons for pyoluterine and 2,4-DAPG using primers shown in Table S2, but many other potential antifungal molecules were confirmed, which we discuss below.

In our study, three nodule endophytes classified on the basis of housekeeping genes to the *P. chlororaphis* species showed antagonism against important fungal phytopathogens: *B. cinerea*, *F. oxysporum*, and *S. sclerotiorum,* known to be responsible for crop diseases and postharvest losses [5,6]. The obtained results indicated that both diffusible and volatile bacterial compounds participated in the mycelial growth inhibition. Two strains designated as 16B1 and 23aP showed similar chromatographic profiles of compounds secreted into the medium. Among them, phenazines were the most abundant group with the highest yield for 2-OH-Phz (above 50%), followed by PCA and 2-OH-PCA. Altogether, they accounted for about 98% of the relative content of identified metabolites. In the case of strain 16A1, a slightly different profile of secondary products was obtained, but still the total relative content of the above-mentioned compounds was very close. The most significant difference was the inverted proportion of PCA and 2-OH-Phz, where the former dominated (almost 60%). In turn, 2-OH-PCA was secreted at the same level by all the isolates. Since the production of phenazines in *P. chlororaphis* strains is under the control of many regulatory genes, the different yield of phenazine derivatives produced by our strains can be related with such mechanisms [20]. The detailed analysis of the LC-MS/MS data revealed that other phenazine derivatives, typical for the biosynthetic pathways of these compounds in *P. chlororaphis* strains [25], were present in the post-culture extracts in the order of relative quantity Phz, 2,8-di-OH-Phz, Phz-1,6-di-COOH, and 6-methyl-PCA.

In most pseudomonas, phenazine biosynthesis genes are arranged in one core operon *phzABCDEFG*, which is responsible for the production of PCA. However, in *P. aeruginosa*, additional phenazine-modifying genes may be present, i.e., *phzM* and *phzS* involved in the production of pyocyanin and *phzH* engaged in the phenazine-1-carboxamide (PCN) production, while *phzO* and *phzH* associated with 2-OH-PCA and PCN synthesis, respectively, may be present in fluorescent pseudomonas [34]. Typically, in a single *P. chlororaphis* strain, PCA is modified to either 2-OH-PCA or PCN [20,35]. In the case of PCN producers, no significant quantities of PCA or other phenazine derivatives are accumulated [35]. Simultaneously, in a single strain with the *phzO* gene, PCA serves as a precursor of both 2-OH-PCA and 2-OH-Phz, being a bright orange pigment [36]. Also, in some *Pseudomonas* strains, the PhzG enzyme can, next to PCA, generate Phz-1,6-di-COOH and unsubstituted phenazine although their yields are very low [24]. In the present study, we confirmed the occurrence of the core operon *phzF* gene and the phenazine-modifying *phzO* gene in the genomes of all the tested strains, which was in agreement with the results of metabolome research and the literature data [20,24,36].

Natural phenazines are heterocyclic compounds substituted at different points around their rings with diverse chemical groups, which influence their different physicochemical properties, thus affecting their biological activities [34]. However, all phenazines are thought to be able to kill off competing fungi through the production of reactive oxygen species. The liberation of specific mixtures of phenazines by *Pseudomonas* strains affects their capacity to suppress fungal disease at many levels as well as a range of susceptible pathogens [24,37]. The results obtained in the present study in the antagonism assays indicated that strains 16B1 and 23aP limited the mycelial growth of all the phytopathogens tested to the same extent, while the biological activity of the 16A1 isolate was significantly lower (Figure 1A). A similar assay was performed by Maddula et al. [38] for closely related *P. chlororaphis* subsp. *aurefaciens* strain 30–84 and indicated that mutual proportions of PCA and 2-OH-PCA were crucial for inhibition of the fungal pathogen *Gaeumannomyces graminis* var. *tritici*. Other studies of the same issue were conducted with the use of *phzO*-producing *P. chlororaphis* GP72 and 06 strains [20]. An additional confirmation of the effectiveness of phenazines in controlling fungal pathogens is the commercialization of pure PCA as an antifungal pesticide registered as “Shenqinmycin” in China in 2011. However, it is known that some pathogens are more fragile to 2-OH-PHZ than PCA [24,39].

Besides phenazines, other fungistatic metabolites were identified in the post-culture extracts of the tested strains: pyrrolnitrin (PRN), lahorenoic acids A and C, cyclic peptides, and 2-acetamidophenol (AAP) [25,40]. Pyrrolnitrin represents phenyl pyrrole antibiotics with broad-spectrum antifungal activity and exerts broad-range cytotoxic effects, e.g., on *S. sclerotiorum*, *B. cinerea*, and some species of the Fusarium genus [41]. It is produced in a small amount, which can be increased under the influence of plant root secretions [42] by many *Pseudomonas*, including these from the group studied. It was established that mutants of *P. chlororaphis* O6 defective in PRN were drastically impaired in the biocontrol of Phytophthora infestans on tomatoes in relation to a WT strain capable of production of both PCA and PRN [43]. In our study, the possibility of pyrrolnitrin production by 16A1, 16B1, and 23aP was confirmed in the comparative metabolomic analysis of all the studied strains. Lahorenoic acid A–C derivatives were reported for the first time in the biocontrol *P. aurantiaca* strain PB-St2 active against Colletotrichum falcatum and later in only few other strains [25,40]. In our study, lahorenoic acid A was present in all our strains with similar abundance, while the highest yield of lahorenoic acid C was provided by 16A1. Among substances produced by *Pseudomonas* spp. and shown to suppress some fungal phytopathogens, there are secretory cyclic peptides. For example, antifungal activity was exhibited by cyclo(L-Pro-L-Val) from *P. aurantiaca* IB5-10 against R. solani, maculosine against Ralstonia solanacearum [44], and cyclo(L-Pro-L-Phe), cyclo(trans-4-hydroxy-L-Pro-L-Leu), cyclo(trans-4-hydroxy-L-Pro-L-Phe), and cyclo(Gly-L-Pro) from *P. fluorescens* against Fusarium moniliforme [45]. Probably, there are a few mechanisms of action of these short cyclopeptides, including disruption of fungal cell membranes, inhibition of the activity of enzymes involved in their cell wall synthesis, and induction of plant defense responses [44]. Cyclic peptides also show antinematode, antibacterial, and antiviral activities [46,47]. In our study, the ethyl acetate extracts of post-cultures of all the strains contained similar levels of cyclo-(L-Pro-L-Val) and maculosine, while the cyclo-(L-Pro-L-Met-diketopiperazine) yield was slightly higher in 16B1 and 23aP, compared to strain 16A1. AAP, first reported in the liquid culture of *P. fluorescens* strain 2–79, was also identified among the diffusible products in the post-culture extracts of our strains. According to previous reports, this compound has antifungal, anti-inflammatory, antitumor, antiplatelet, and antiarthritic activities. The metabolomic data analysis revealed quite a high yield of AAP, in comparison to other fungistatic metabolites excluding phenazines (Figure 2, Table S1). This is related to the fact that AAP shares the same biosynthetic route with PCA [24].

Limitation of the growth of fungal phytopathogens with participation of biocontrol organisms also takes place through competition, which is achieved mainly with the participation of extracellular siderophores demonstrating a strong iron-repressed antagonism in vitro [19]. Since iron is vital to the growth, virulence, and even survival of pathogens, its depletion results in inhibition of mycelial growth, especially in low-iron conditions. *P. chlororaphis* strains commonly liberate pyoverdine siderophores with high-iron affinity and achromobactin with low-iron affinity into the environment [20]. The present results confirm that strains 16A1, 16B1, and 23aP are very good producers of these iron chelators (Figure 3D; *p* > 0.05), and, similar to many other pseudomonads, they are capable of synthesis of pyoverdine (Figure 3C).

The mixture of VOCs liberated by microorganisms into the soil play a significant role in the control of phytopathogen population overgrowth in the rhizosphere environment [48]. Their emission carried out by some plant tissue-associated bacteria provides effective protection to host endogenously [49]. In the current study, the VOCs liberated by strains 16A1, 16B1, and 23aP were highly effective against *B. cinerea* [29], while *S. sclerotiorum* appeared fully resistant to all these compounds. In turn, as indicated by the literature data, this fungus was susceptible to VOCs from *P. chlororaphis* subsp. *aureofaciens* SPS-41 and *P. chlororaphis* PA23 [50,51]. Therefore, it seems that the composition of the mixture of volatile compounds is crucial for effective antifungal action. Among VOCs, HCN is thought to be one of the strongest biocontrol agents. The mechanism of cyanide action is hypothesized to rely on binding to the ferric ions in cytochrome oxidase in an electron transport chain and other metalloenzymes, which can block the reduction of oxygen to water [52]. The data obtained in our bioassays showed that all the *P. chlororaphis* isolates were capable of HCN production. However, using primers mentioned in Table S2, no amplicons of *hcn* and *hcnBC* genes involved in the HCN biosynthetic pathways were achieved. Taking into account these considerations, the aim of our future study will be to carry out a thorough chemical analysis of VOCs and molecular pathways of their synthesis.

To sum up, the overall data analysis implied that the level of biosynthesis of individual metabolites or their mutual proportions might have influenced the scope of antifungal activity of our strains. In support of this hypothesis, the review by Price-Whelan et al. [34] provides more evidence.

## 4. Materials and Methods

### 4.1. Bacterial Strain Isolation and Media

*Pseudomonas chlororaphis* strains 16A1 (16A1), 16B1 (16B1), and 23aP (23aP) were isolated from root nodules of *Chamaecytisus albus* (Hacq.) Rothm. The host plant was obtained from a locality near Hrubieszów town in Poland [21]. The isolation of endophytes was preceded by surface disinfection of the roots carried out by sequential washing in sterile water, 70% ethanol (*v*/*v*) for 2 min, a sodium hypochlorite solution for 5 min, 70% ethanol (*v*/*v*) for 1 min, and, lastly, several times with sterile water to remove epiphytes [53]. The suspension obtained from crushed nodules [54] was streaked on yeast mannitol medium (YEM) and incubated at 28 °C for 3–5 days. The cultivable isolates were purified several times using the streak plate technique on YEM agar, followed by preservation in YEM with 50% (*v*/*v*) glycerol and storing at −20 °C. Between the experiments, the strains were stored on 79CA medium.

The fungi used in the research—*Fusarium oxysporum* 10Fo01, *Botrytis cinerea* 10Bc01, and *Sclerotinia sclerotiorum* 10Ss01—came from a collection of the Warsaw University of Life Sciences. Between the experiments, the strains were stored on Sabouraud dextrose agar.

The strains were deposited in the bank collection of the Department of Genetics and Microbiology of Maria Curie-Sklodowska University in Lublin, Poland.

Standard succinate medium (SSM) had the following composition (in g·L^–1^): K_2_HPO_4_—6.0, KH_2_PO_4_—3.0, (NH_4_)_2_SO_4_—1.0, MgSO_4_—0.2, and succinic acid—4.0. Before sterilization, the pH of the medium was adjusted to pH 6.8 using 2 M NaOH.

### 4.2. Phylogenetic Analysis of Selected Strains and Molecular Identification of Genes Commonly Associated with Biological Control Activity

The DNA from strains 16A1, 16B1, and 23aP was isolated according to a method described by Pitcher et al. [55]. For determination of the genomic DNA purity and concentration, measurements with NanoDrop™ 2000/2000c (Thermo Fisher Scientific, Wilmington, DE, USA) were carried out. The extracted DNA was stored at –20 °C.

The taxonomic position of strain 23aP was determined based on MLSA [22]. The position of strains 16A1 and 16B1 was determined based on comparative analysis of housekeeping genes *recA* and *gyrB* using primers in conditions described by Karaś et al. [22].

PCR was performed using specific primers to detect *phl, rzxB, plt*, and *hcnBC* genes (Table S2) in conditions described by Gomez-Lama et al. (2018) [56]. In the analysis of strains 16A1, 16B1, and 23aP, primers for *prnB, hcn, plt, prnC, phzF*, and *phzO* genes, designed based on the sequences of the corresponding genes of the reference strains available in the GenBank database (Table S2), were also used. PCR of products obtained only for three analyzed genes, i.e., *phzF, phzO*, and *prnC*, and the phylogenetic analysis of the isolates were performed using the obtained sequences. All the sequences analyzed were amplified in PCR using a Taq PCR Master Mix (2×) kit (EURx, Gdańsk, Poland) according to the manufacturer’s instruction. A total of 100 ng of template DNA and 0.4 mM of forward and reverse primers were added to the reaction mixture. PCR amplifications were performed using a thermal cycler (Biometra TProfessional basic thermocycler gradient) in the following conditions: initial denaturation at 94 °C for 3 min, followed by 35 cycles of 94 °C for 45 s, 52 °C for 1 min, and 72 °C for 1.5 min, with a final extension step at 72 °C for 7 min.

The amplicons were purified using a Clean-Up purification kit (A&A Biotechnology, Gdańsk, Poland) and sequenced with a BigDyeTM Terminator Cycle sequencing kit (Applied Biosystems, Foster City, CA, USA). The samples were sequenced at an external company (Genomed SA, Warsaw, Poland). Sequences were searched against existing references in the GenBank database using the BLAST+ 2.16.0 tool. The GeneDoc program was used for visualization of the alignment performed using ClustalX2.1 program [57]. A phylogenetic tree was generated based on the achieved sequence set using MEGA11 software with the Neighbor-Joining (NJ) method [58]. The two-parameter Kimura model was used as a nucleotide substitution model. jModelTest selected the best-fitting evolutionary model for each tested gene [59]. The statistical significance of the tree was evaluated with the bootstrap test (1000 replicates). The phylogenetic tree was represented in TreeView program [60].

### 4.3. Beneficial Features of Plant Growth Promotion (PGP)

The PGP properties of strains 16A1, 16B1, and 23aP were studied. The ability to produce IAA was checked using the method described by Tiwari et al. (2016) [61]. The capacity to solubilize phosphates was tested on tricalcium phosphate agar (TCP) [62]. Cellulolytic and proteolytic activities were determined on modified Congo red cellulose agar [62,63] and milk agar [64], respectively. The production of HCN was carried out according to the method described by Lorck [65].

The production of siderophores was screened using the Chrome Azurol S (CAS) assay [66] after incubation of bacterial cultures at 28 °C for 4 days. The diameter of colored zones was used to determine the siderophore production index (SPI) as SPI = diameter of orange halo zone/diameter of colony.

Catechol siderophores produced by the tested strains were quantitatively tested using the Arnow test [67]. To study the in vitro production of catechol-type phenolates, the isolates were grown in standard succinate medium (SSM) (pH 6.8) for 72 h at 28 °C on a rotary shaker at 120 RPM. After this time, the cultures were centrifuged, and the supernatants were acidified to pH 2.0 and extracted three times with an equal volume of ethyl acetate. After evaporation of the organic solvent, the samples were diluted in ethanol (1 mL). Then, siderophores were measured in ethanolic solutions using Hathway’s ferric chloride–ferricyanide reagent [68]. For the assay, 125 µL of the reagent was added to each 500 µL sample, and after 5 min of incubation, absorbance was determined at 560 nm (*A*_560_) for salicylates and at 700 nm (*A*_700_) for dihydroxyphenols. Dihydroxybenzoic acid (2,3-DHBA) and salicylic acid (SA) were used as standards for calibration curves.

For evaluation of the pyoverdine concentration, UV–VIS spectroscopy was used in the wavelength range of 380–400 nm, corresponding to its maximum absorbance. The registered data were used to determine the pyoverdine concentration using the Lambert–Beer law with the molar extinction coefficient (ε) of 16,000 Lmol^−1^cm^−1^ [69]. To avoid Fe contamination, all glassware was soaked with 6 M HCl overnight and then washed with ultrapure water (Milli-Q system, Bedford, MA, USA).

### 4.4. In Vitro Antagonistic Activity against Fungi

To evaluate the in vitro antagonistic activity of strains 16A1, 16B1, and 23aP against *Fusarium oxysporum* 10Fo01, *Botrytis cinerea* 10Bc01, and *Sclerotinia sclerotiorum* 10Ss01, the disc diffusion method was used [70]. Plates with 79CA agar medium inoculated with 100 µL of a suspension of a single bacterial strain in sterile saline (OD_600_ = 0.2) were incubated at 28 °C for 5 days in the dark. After the incubation period, a 5 mm diameter agar disc, aseptically cut from a particular bacterial colony, was placed 22.5 mm from the edge of the potato dextrose agar [71] plate (PDA plate; Ø 90 mm). Simultaneously, a 5 mm diameter agar disc aseptically cut from a 7-day-old colony of appropriate fungi was placed at the same distance from the opposite edge of plate. The incubation was carried out at 28 °C for five days, followed by measurement of fungal inhibition zones around bacteria. PDA plates inoculated by the tested fungi without bacterial discs were used as a control. The incubation was carried out at 28 °C for five days, i.e., a period necessary for full overgrowth of mycelia on the control plates. Next, the diameters of the fungal inhibition zones around the bacterial discs were measured. The antifungal activity was calculated as the percent of mycelial growth inhibition (PGI_1_) using the following equation: PGI_2_ (%) = (Dc − Dt)/Dc × 100%, where Dc represents the diameter of the control fungal colony, and Dt represents the diameter of the treatment colony.

### 4.5. Antifungal Activity of Volatile Compounds (VOCs) from Endophytic Bacteria in an In Vitro Assay

The antifungal activity of VOCs produced by endophytic bacteria was investigated using a two-sealed base plate method. One base plate containing 15 mL of 79CA agar medium was inoculated with 100 µL of a single bacterial suspension in sterile saline (OD_600_ = 0.2). Then, a 5 mm diameter disc with mycelium was cut from a 5-day-old fungal colony and transferred into another plate containing 15 mL of PDA medium. The two base plates were tightly sealed with microplate sealing film and then incubated at 28 °C for 5 days in the dark. Non-inoculated plates were used as a control. Volatile antifungal activity was calculated as the percent of mycelial growth inhibition (PGI_2_) using the following equation: PGI_2_ (%) = (Rc − Rt)/Rc × 100, where Rc represents the radius of the control fungal colony, and Rt represents the radius of the treatment colony [7].

### 4.6. Extraction and Identification of Antifungal Metabolites in Cell-Free Extracts

For the production of secondary metabolites, 300 mL flasks containing 79CA medium (80 mL) were inoculated with overnight cultures of 16A1, 16B1, and 23aP at OD_600_ = 0.56–0.6 (5% *v*/*v*). Incubation was carried out for 6 days at 28 °C with shaking at 165 RPM. To control the production of phenazines, 300 µL portions of cultures were collected daily, and after centrifugation, the absorbance of cell-free supernatants was measured at 367 nm, i.e., the maximum absorbance, for PCA and 490 nm for 2-OH-PHZ versus the control (non-inoculated 79CA). After the stipulated fermentation time, the collected culture broths were adjusted to pH 2.0 with 6 M HCl and extracted three times with an equal volume of ethyl acetate under vigorous shaking. The organic phases were combined and evaporated under vacuum pressure at 40 °C. Cell-free extract metabolites were dissolved in methanol, and the metabolites were used for HPTLC, GC-MS, and LC-MS/MS analyses.

#### 4.6.1. HPTLC Analysis

For separation of metabolites from cell-free extracts, high-performance thin layer chromatography (HPTLC) was performed. Separation was carried out on a silica gel plate (10 × 10 cm; 60 F_254_, Merck, Darmstadt, Germany) with chloroform/acetic acid (49:1; *v*/*v*) as the developing mixture [26]. After the first separation, the plates were dried and re-chromatographed in the same conditions and in the same direction to better separate the components of the extract. Visualization was carried out in visible and under short (254 nm) and long (365 nm) UV light wavelengths. The extract from bacteria-free medium was used as a negative control. It was prepared according to the same procedure and analyzed as the experimental samples.

#### 4.6.2. GC-MS

For further identification, dried methanol extracts from cell-free cultures of 16A1, 16B1, and 23aP dissolved in chloroform/MeOH (2:1, *v*/*v*) were identified without derivatization using a gas chromatograph (Agilent Technologies, Wilmington, DE, USA, instrument 7890A) coupled with a mass-selective detector (Agilent Technologies, USA, MSD 5975C, inert XL EI/CI). The GC-MS system was equipped with an HP-5MS capillary column (30 m × 0.85 mm) with helium as a carrier gas (1 mL × min ^−1^). The analyses were carried out using the EI mode (70 eV) with the following temperature program: 150 °C (5 min), then up to 310 °C with an increase of 5 °C per minute and 10 min at 310 °C.

#### 4.6.3. LC-MS and LC-MS/MS Analyses

Sample solutions were injected onto an Eclipse Plus C18 RRHD column (2.1 × 100 mm, 1.8 µm; Agilent) and separated by reverse-phase liquid chromatography using a Vanquish UPLC system (Thermo) at a flow rate of 0.4 mL/min at 40 °C. The UPLC system was coupled to an Orbitrap Exploris 480 mass spectrometer (Thermo) equipped with an OptaMax NG API source. The mobile phases were water/0.1% formic acid (A) and acetonitrile/0.1% formic acid (B). The column was equilibrated for 3 min at 5% B; after which, the metabolites were separated on the column with a 17 min gradient (0 min, 5% B; 0–15 min, 5–100% B; 15–17 min, 100% B). The following ion source parameters were set: spray voltage in positive mode, 2500V; capillary temperature, 325 °C; vaporizer temperature, 350 °C; sheath gas, 50; auxiliary gas, 10; and sweep gas, 1. The mass spectrometer operated in data-dependent acquisition mode. The MS survey scan was carried out over the mass range of 80–800 *m*/*z* and RF Lens set to 50%. Data were recorded in the profile mode and at 120K resolution. The AGC target was set to ‘standard’ with 1 microscan and maximum injection time set to ‘auto’. The following parameters were applied for DDA processing: the MIPS mode was set to ‘small molecule’ with relaxed restrictions, the intensity threshold was set to 50,000, the include charge state was set to 1, the dynamic exclusion n times was set to 1 with 10 s duration and +/− 10 ppm mass tolerance, and the custom targeted inclusion list was set for the expected metabolites. The following parameters were used for the MS/MS experiments: the isolation window was set to 2 *m*/*z* for quadrupole and resolution was set at 15K for orbitrap in profile mode, normalized stepped collision energy was applied (30, 50, 80%), AGC was set to standard with 1 microscan, and maximum injection time was set to 100 ms. The total cycle time was set to 0.8 s. Each sample was injected one time. The mass spectrometer was operated using Xcalibur 4.6.67.17 software (Thermo). The data were processed using Freestyle 1.8 SP2 QF1 software (Thermo). The relative contents of the metabolites were calculated according to corrected mass spectra peak areas and presented as the heat map created using Microsoft Office Excel 2010 for Windows software (Microsoft Corp., Redmond, WA, USA).

### 4.7. Statistical Analyses

The experiments were performed independently three times, and each experiment comprised two samples unless otherwise stated. The values obtained were submitted to statistical analyses performed with GraphPad Prism 6.0 software (GraphPad, La Jolla, CA, USA) and presented as means (±SD) of three independent experiments. The significance level of *p*  <  0.05 was established using one-way analysis of variance (ANOVA) or Student’s *t*-test.

### 4.8. Accession Numbers

The GenBank accession numbers for the sequences determined in this work are PQ058685, PQ058686, PQ058687, PQ058688, PQ058689, PQO58690, PQO58691, PQO58692, PQO58693, PQ095915, PQ095916, PQ095917, and PQ095918. The accession numbers of the reference strains are given in phylograms.

## 5. Conclusions

In the current study, we profiled different secondary metabolites produced by endophytic strains of *P. chlororaphis* with antagonistic potential against three phytopathogenic fungi of great importance in the agriculture and food preservation industries. Conclusively, the detailed biological, metabolomic, and genetic analyses confirmed that strains 16A1, 16B1, and 23aP might have potential as biofertilizers, biocontrol agents, and biological fumigants, as they produce a broad spectrum of phenazine derivatives, pyrrolnitrin, VOCs, hydrolytic enzymes, and siderophores, which altogether can ameliorate or reduce deleterious effects of fungal pathogens on plants. However, further in planta work is needed to ascertain that these isolates have biotechnological and potential commercial applications.

The results obtained may also help to preserve biodiversity and support the protection of *Ch. albus*, an endangered plant placed on red lists and in red books.

## Figures and Tables

**Table 1:** PGP traits of *P. chlororaphis* strains.

Strain	Phosphate Solubilization	Cellulolytic Activity	Proteolytic Activity	IAA Production
16A1	+	+	+	ND
16B1	+	+	+	+
23aP	+ ^a^	+ ^a^	+ ^a^	+

+ present; ND—not detected; and ^a^—data published in [22].

**Table 2:** Metabolites identified in extracts from cell-free supernatants of 16A1, 16B1, and 23aP cultures using LC-MS/MS analysis. Their structures were established by comparing spectrometric data with those in the literature [25]. Chromatograms and fragmentation patterns for all compounds are presented in Supplementary Materials (Figures S2 and S3).

Metabolites	R*_t_* (min)	Chemical Formula	Monoisotopic *m*/*z* [M + H]	Observed peak *m*/*z* [M + H]
hexahydroquinoxaline-1,4-dioxide	2.22	C_8_H_12_N_2_O_2_	169.0977	169.0971
maculosine [cyclo-(L-Pro-L-Tyr)]	2.49	C_14_H_16_N_2_O_3_	261.1239	261.1231
cyclo-(L-Pro-L-Val)	2.75	C_10_H_16_N_2_O_2_	197.1290	197.1284
cyclo-(L-Pro-L-Met-diketopiperazine)	2.86	C_10_H_16_N_2_O_2_S	229.1011	229.1003
2-acetamidophenol	3.13	C_8_H_9_NO_2_	152.0712	152.0706
6-methyl-PCA	4.43	C_14_H_10_N_2_O_2_	239.0821	239.0814
2,8-di-OH-Phz	4.45	C_12_H_8_N_2_O_2_	213.0664	213.0656
2-OH-Phz	5.47	C_12_H_8_N_2_O	197.0715	197.0709
Phz-1,6-di-COOH	6.57	C_14_H_8_N_2_O_4_	269.0562	269.0555
PCA	6.76	C_13/_H_8_N_2_O_2_	225.0664	225.0657
2-OH-PCA	6.92	C_13_H_8_N_2_O_3_	241.0613	241.0606
lahorenoic acid A	7.01	C_16_H_20_O_3_	261.1491	261.1483
Phz	7.05	C_12_H_8_N_2_	181.0766	181.0759
lahorenoic acid C	7.70	C_16_H_20_O_2_	245.1542	245.1535
pyrrolnitrin	8.94	C_10_H_6_Cl_2_N_2_O_2_	256.9885	256.9876

## Data Availability

The data presented in this study are contained within the article or Supplementary Materials.

## Supplementary Materials

The following supporting information can be downloaded at https://www.mdpi.com/article/10.3390/molecules29184370/s1, Figure S1. HPTLC chromatograms of ethyl acetate extracts of cell-free *Pseudomonas chlororaphis* culture supernatants. Separation was performed with chloroform/acetic acid (49:1; *v*/*v*) as the developing mixture. The area containing the spots with the greatest difference in intensity is marked with a red frame. Figure S2. Chromatograms of metabolites of cell-free supernatants of 16A1, 16B1, and 23aP *P. chlororaphis* cultures registered in (A) GC-MS analysis and (B) LC-MS analysis. K—negative control; ethyl acetate extract obtained from bacteria-free 79CA medium. Table S1. Relative contents of metabolites in LC-MS analysis calculated according to corrected mass spectra peak areas. Figure S3. Secondary metabolites identified in cell-free supernatants of 16A1, 16B1, and 23aP *P. chlororaphis* cultures. (I) Ionograms for particular compounds, and (II) MS or MS/MS scans registered for particular compounds in LC-MS/MS analysis used for their identification. Figure S4. The maximum likelihood phylogenetic trees based on 701 bp fragments of the *phzO* gene (A), 694 bp fragments of the *prnC* gene (B), and 764 bp fragments of the *phzF* gene constructed using the Neighbor-Joining (NJ) method [58]. Bootstrap values [%] higher than 50 are shown next to the branches. The scale bar represents 0.05 substitutions per nucleotide position. Table S2. Primers used in this study.
