# Supplementary material for: Profiling Metabolites with Antifungal Activities from Endophytic Plant-Beneficial Strains of Pseudomonas chlororaphis Isolated from Chamaecytisus albus (Hack.) Rothm
Source: Molecules. 2024 Sep 14;29(18):4370. doi: 10.3390/molecules29184370 (PMC11433735; doi:10.3390/molecules29184370)
Supplement: Supplementary file 1 [file molecules-29-04370-s001.zip › molecules-3164622-supplementary.pdf]

Supplementary materials

Profiling metabolites with antifungal activities from endophytic plant-beneficial strains of *Pseudomonas chlororaphis* isolated from *Chamaecytisus albus* (Hack.) Rothm.

Wojciech Sokołowski[1], Monika Marek-Kozaczuk[1], Piotr Sosnowski[2], Ewa Sajnaga[3], Monika Elżbieta Jach[4], and Magdalena Anna Karaś*[1]

2.3. The analysis of diffussible metabolites

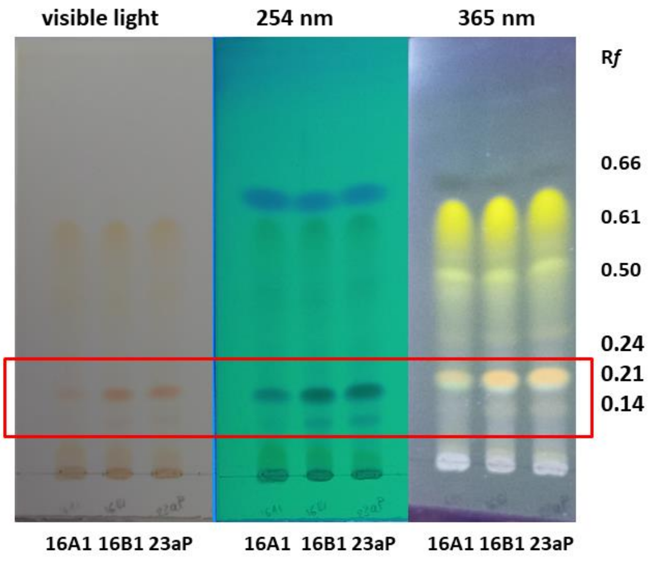

Figure S1. HPTLC chromatograms of ethyl acetate extracts of cell-free *Pseudomonas chlororaphis* culture supernatants. Separation was performer with twice with chloroform/acetic acid (49:1; *v/v*) as the developing mixture. The area containing the spots with the greatest difference in intensity is marked with a red frame. 16A1, 16B1, 23aP – strains tested

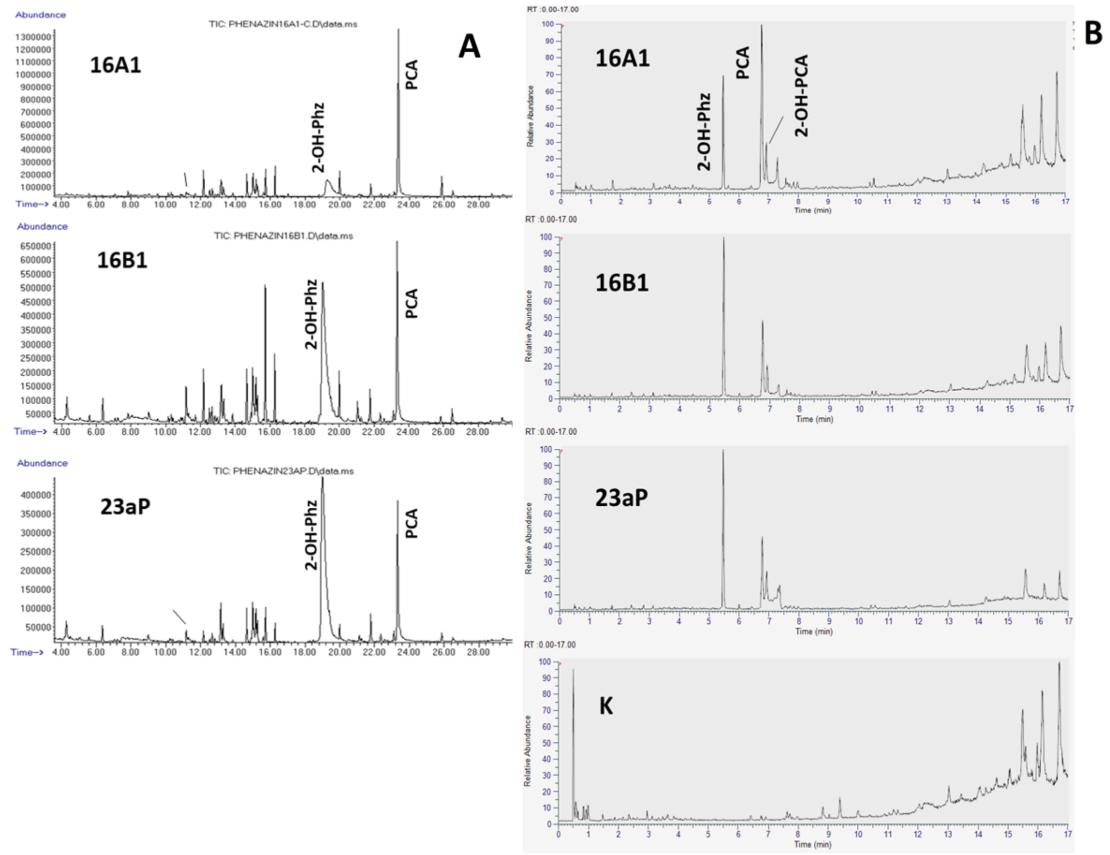

Figure S2. Chromatograms of metabolites obtained of cell-free supernatants of 16A1, 16B1, and 23aP *P. chlororaphis* cultures registered in (A) GC – MS analysis, (B) LC – MS analysis. K – negative control; ethyl acetate extract obtained from bacteria-free 79CA medium. Abbreviations: 2-OH-Phz – 2-hydroxyphenazine, PCA – phenazine-1-carboxylic acid, 2-hydroxy-1-carboxylic acid.

Table S1. The relative contents of metabolites in LC – MS analysis calculated according to corrected mass spectra peak areas.

Metabolites	Relative content (%)		
	**16A1**	**16B1**	**23aP**
hexahydroquinoxaline-1,4-dioxide	0.024	0.008	0.006
maculosin [cyclo-(L-Pro-L-Tyr)]	0.116	0.085	0.075
cyclo-(L-Pro-L-Val)	0.203	0.144	0.140
cyclo-(L-Pro-L-Met-diketopiperazine)	0.012	0.010	0.012
2-acetamidophenol	0.610	0.685	0.668
6-methyl-PCA	0.013	0.025	0.023
2,8-di-OH-Phz	0.262	0.314	0.160
2-OH-Phz	28.318	**52.556**	**56.369**
Phz-1,6-di-COOH	0.198	0.111	0.144
PCA	**58.210**	34.778	29.493
2-OH-PCA	11.308	10.776	12.146
lahorenoic acid A	0.236	0.100	0.118
phenazine	0.289	0.168	0.186
lahorenoic acid C	0.194	0.242	0.248
pyrrolnitrin	0.001	0.003	0.002

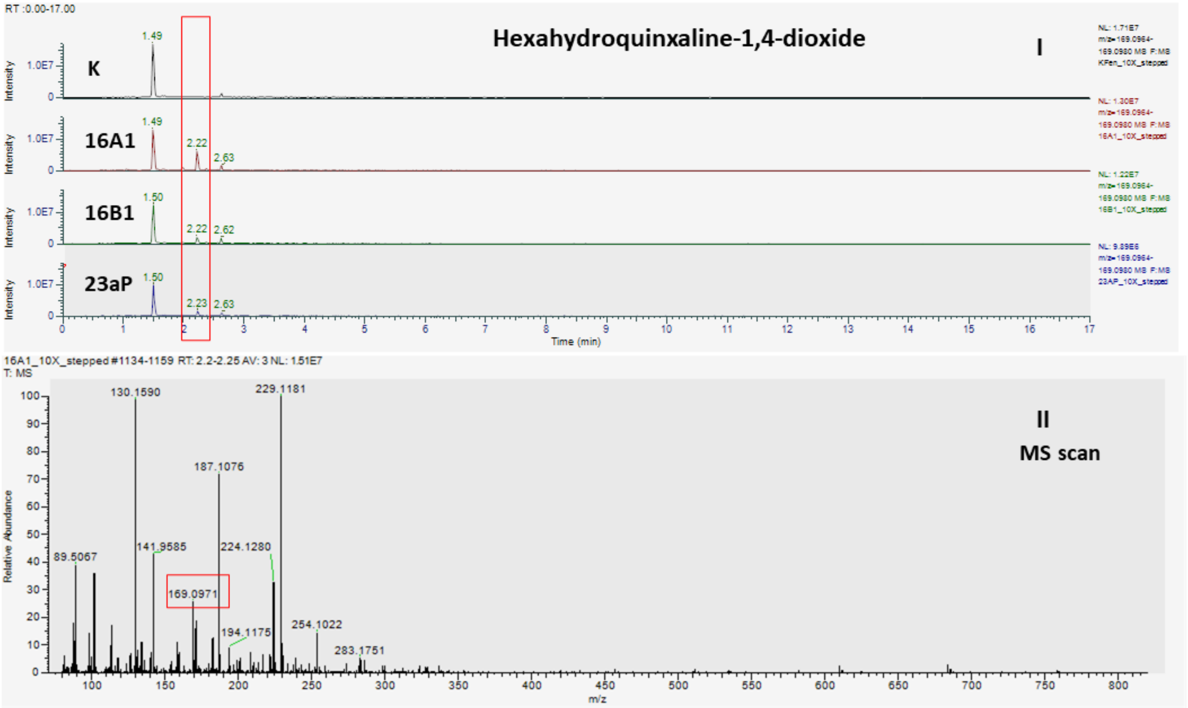
RT :0.00-17.00
Hexahydroquinxaline-1,4-dioxide
I
K
16A1
16B1
23aP
Time (min)
16A1_10X_stepped #1134-1159 RT: 2.2-2.25 AV: 3 NL: 1.51E7
T: MS
II
MS scan
130.1590
229.1181
187.1076
89.5067
141.9585
224.1280
169.0971
194.1175
254.1022
283.1751
Relative Abundance
m/z

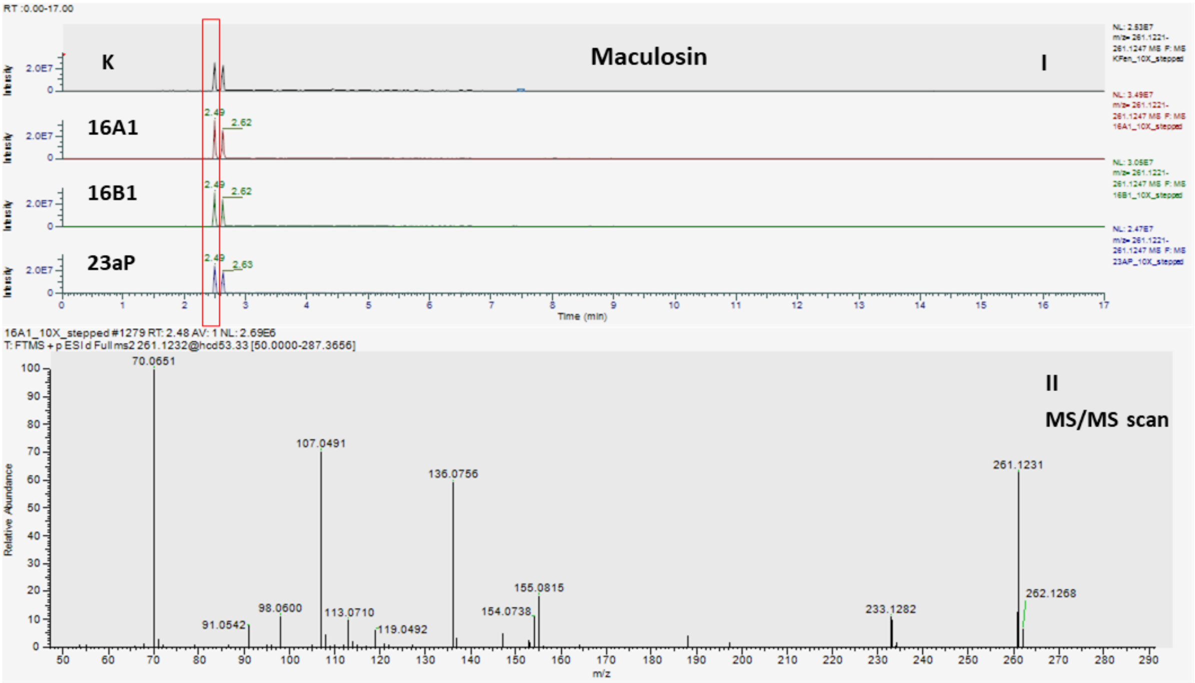
RT :0.00-17.00
Maculosin
I
K
16A1
16B1
23aP
Time (min)
16A1_10X_stepped #1279 RT: 2.48 AV: 1 NL: 2.69E6
T: FTMS + p ESI d Full ms2 261.1232@hcd53.33 [50.0000-287.3656]
II
MS/MS scan
70.0651
107.0491
136.0756
261.1231
155.0815
262.1268
154.0738
233.1282
98.0600
113.0710
91.0542
119.0492
Relative Abundance
m/z

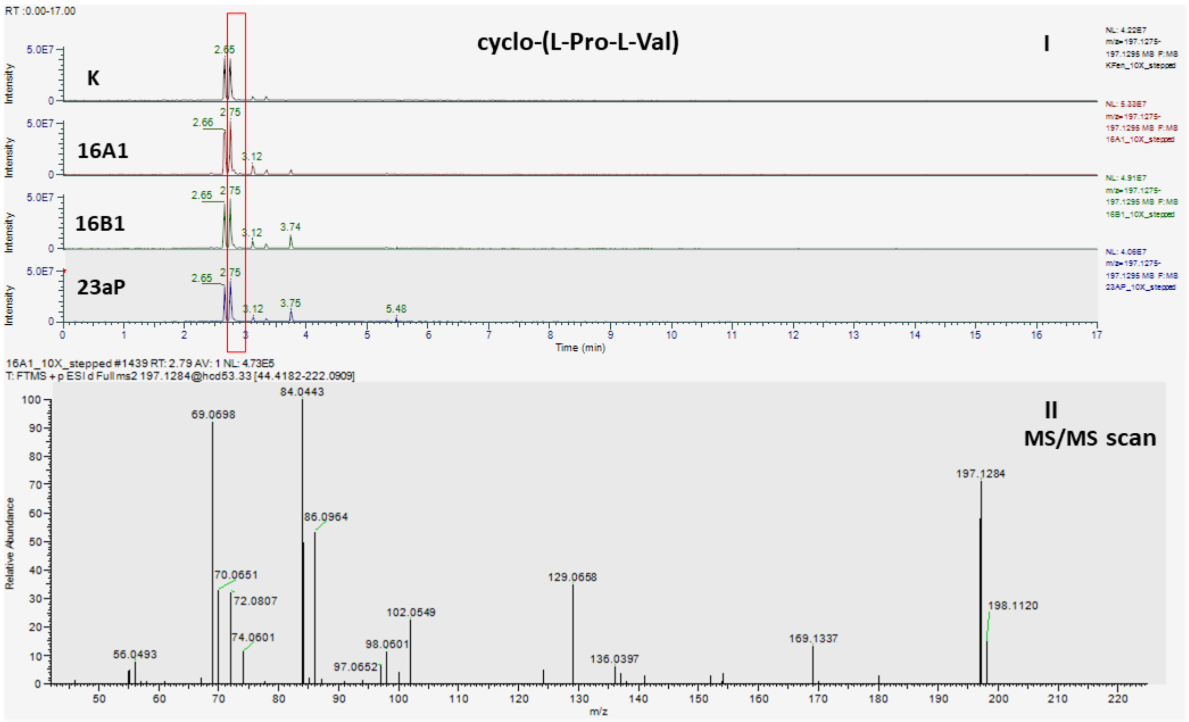

cyclo-(L-Pro-L-Val)
I
K
16A1
16B1
23aP
Time (min)
16A1_10X_stepped #1439 RT: 2.79 AV: 1 NL: 4.73E5
T: FTMS + p ESI d Full ms2 197.1284@hcd53.33 [44.4182-222.0909]
II
MS/MS scan
84.0443
69.0698
197.1284
86.0964
70.0651
72.0807
74.0601
56.0493
97.0652
98.0601
102.0549
129.0658
136.0397
169.1337
198.1120
Relative Abundance
m/z

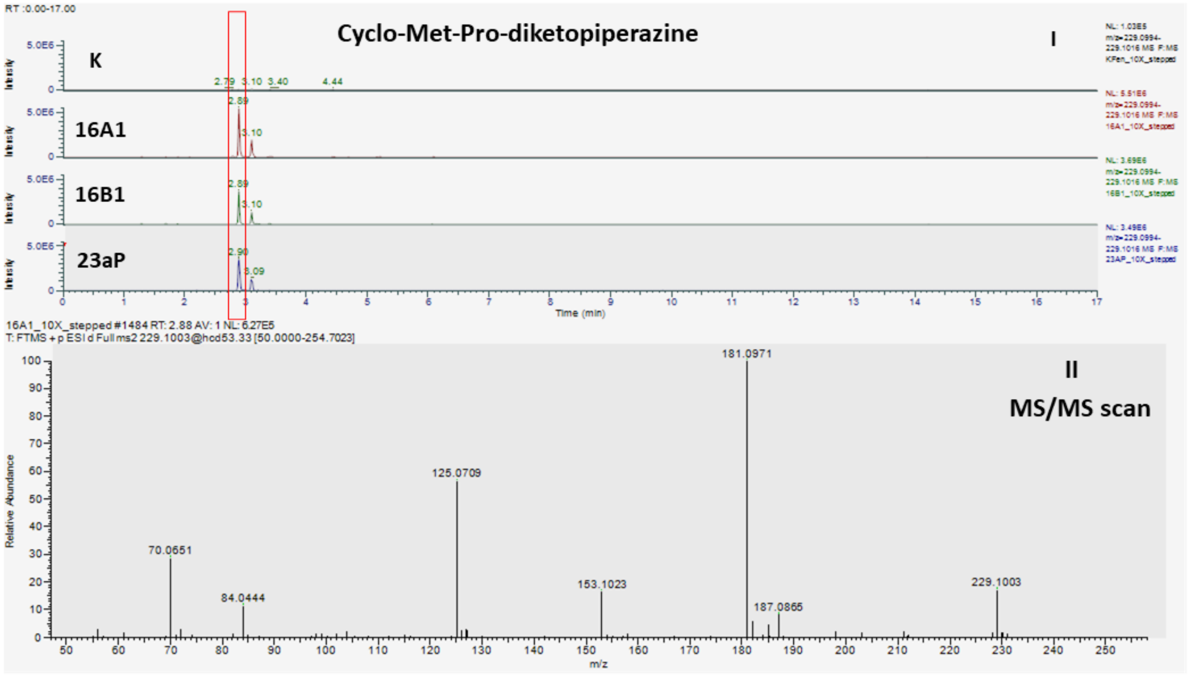

Cyclo-Met-Pro-diketopiperazine
I
K
16A1
16B1
23aP
Time (min)
16A1_10X_stepped #1484 RT: 2.88 AV: 1 NL: 6.27E5
T: FTMS + p ESI d Full ms2 229.1003@hcd53.33 [50.0000-254.7023]
II
MS/MS scan
181.0971
125.0709
70.0651
84.0444
153.1023
187.0865
229.1003
Relative Abundance
m/z

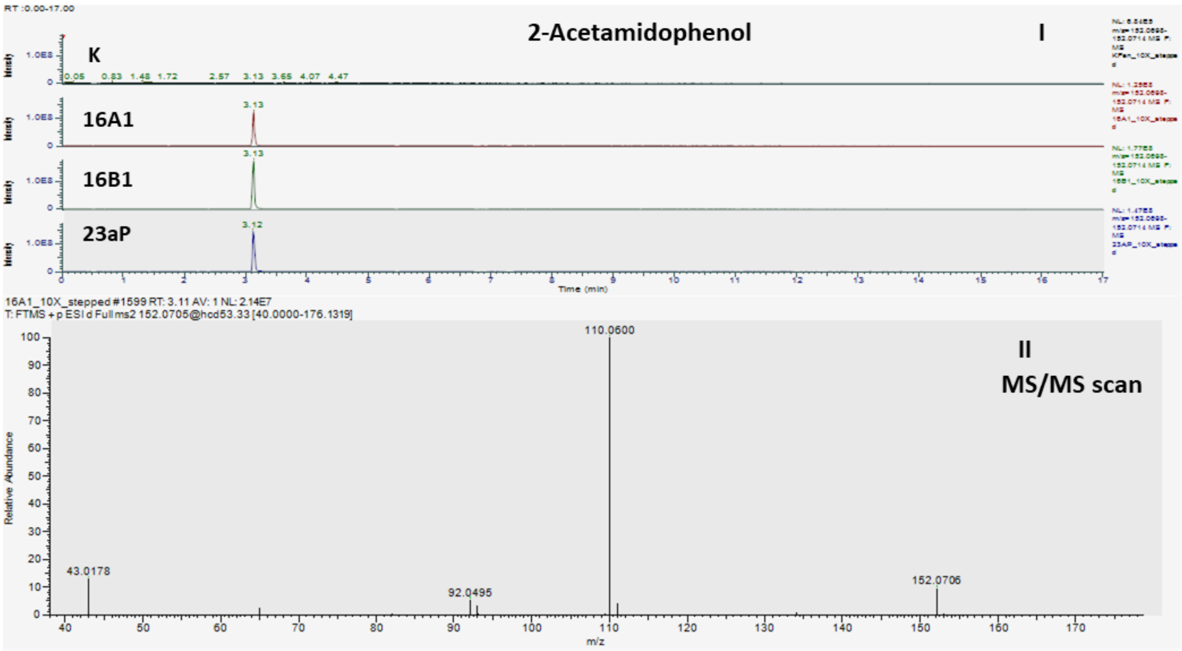
2-Acetamidophenol
I
K
16A1
16B1
23aP
16A1_10X_stepped #1599 RT: 3.11 AV: 1 NL: 2.14E7
T: FTMS + p ESI d Full ms2 152.0705@hcd53.33 [40.0000-176.1319]
110.0600
II
MS/MS scan
43.0178
92.0495
152.0706
Relative Abundance
m/z
Time (min)

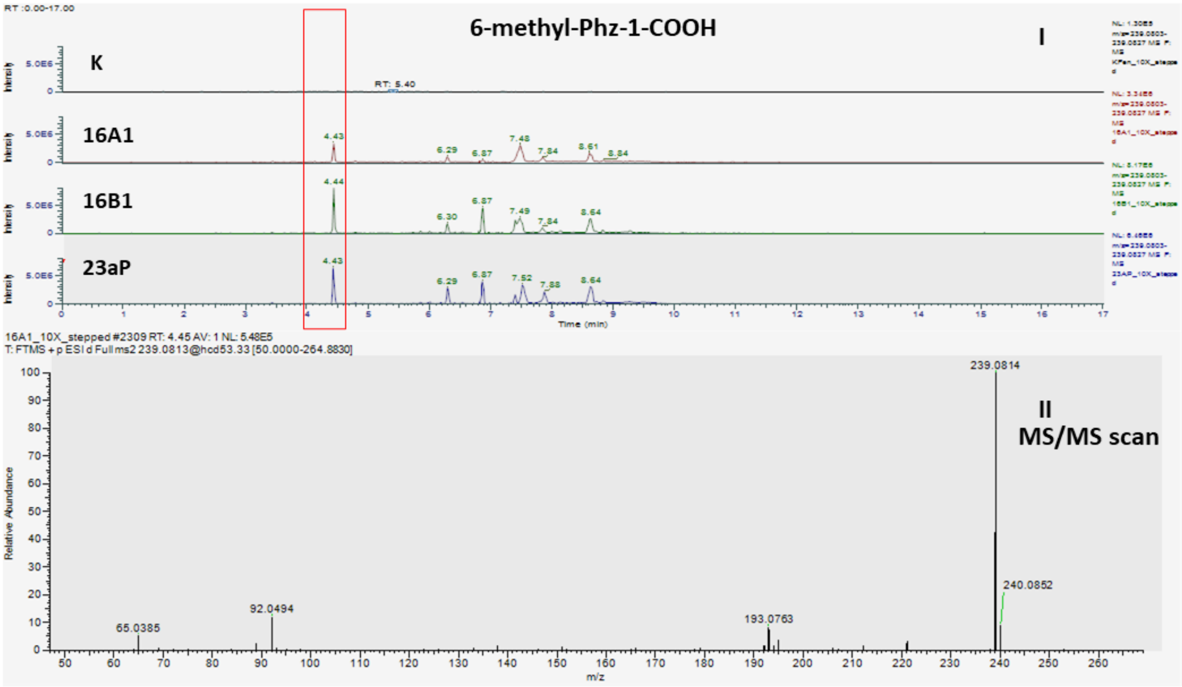
6-methyl-Phz-1-COOH
I
K
16A1
16B1
23aP
16A1_10X_stepped #2309 RT: 4.45 AV: 1 NL: 5.48E5
T: FTMS + p ESI d Full ms2 239.0813@hcd53.33 [50.0000-264.8830]
239.0814
II
MS/MS scan
240.0852
193.0763
92.0494
65.0385
Relative Abundance
m/z
Time (min)

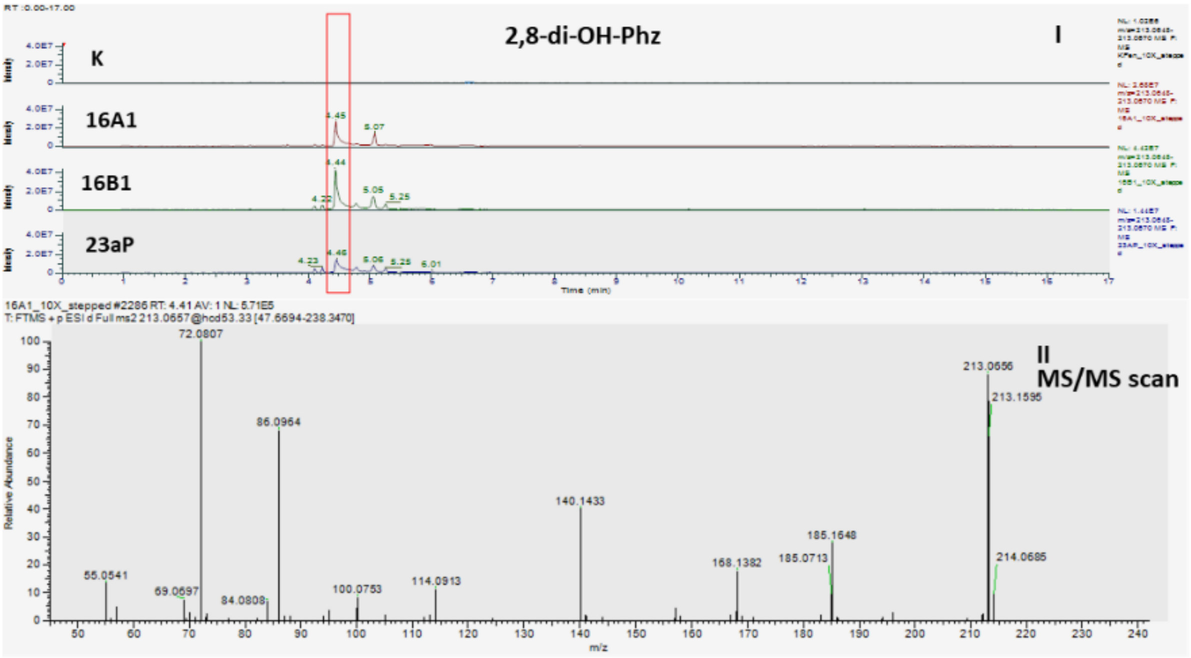

RT :0.00-17.00
2,8-di-OH-Phz
I
K
16A1
16B1
23aP
4.45
5.07
4.44
5.06
5.25
4.46
6.01
Time (min)
16A1_10X_stepped #2286 RT: 4.41 AV: 1 NL: 5.71E5
T: FTMS + p ESI d Full ms2 213.0657@hcd53.33 [47.6694-238.3470]
II
MS/MS scan
72.0807
86.0964
140.1433
185.1648
168.1382
185.0713
213.0656
213.1595
214.0685
55.0541
69.0697
84.0808
100.0753
114.0913
Relative Abundance
m/z

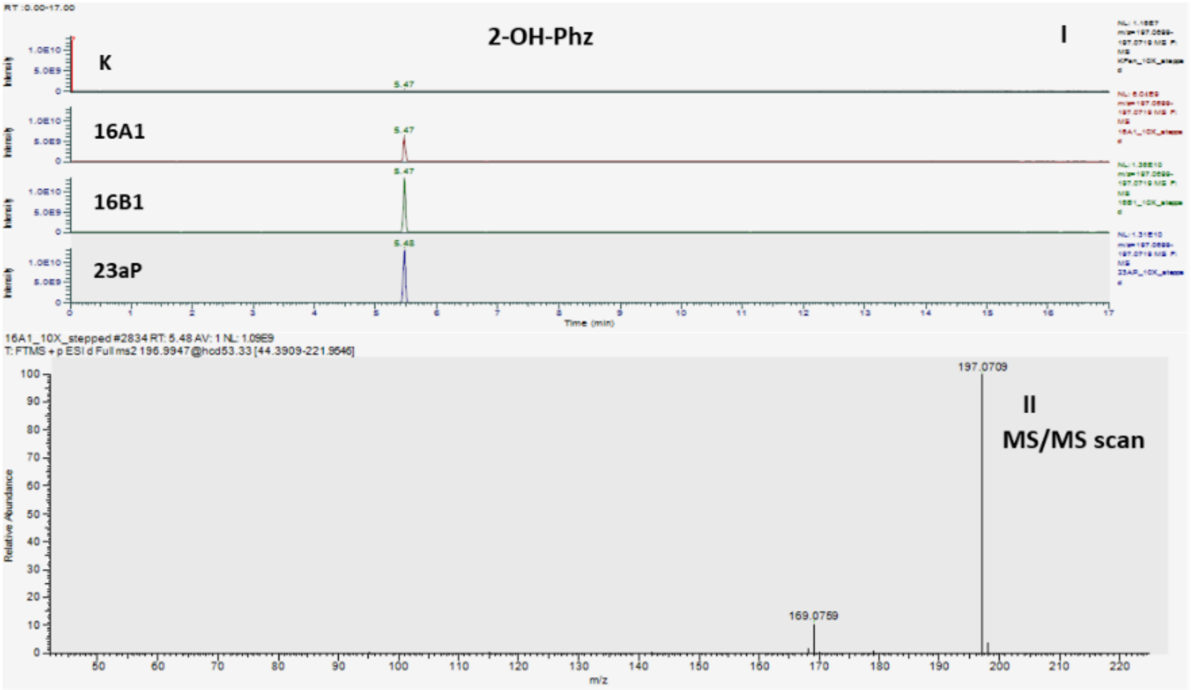

RT :0.00-17.00
2-OH-Phz
I
K
16A1
16B1
23aP
5.47
5.48
Time (min)
16A1_10X_stepped #2834 RT: 5.48 AV: 1 NL: 1.09E9
T: FTMS + p ESI d Full ms2 196.9947@hcd53.33 [44.3909-221.9546]
II
MS/MS scan
197.0709
169.0759
Relative Abundance
m/z

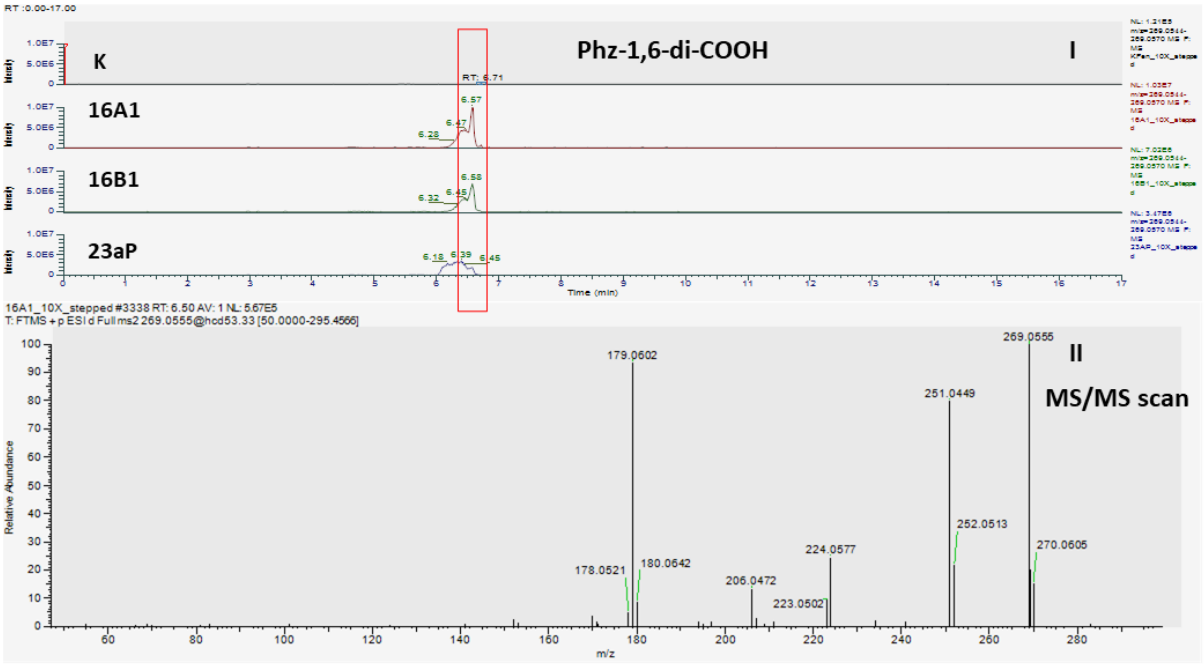
RT :0.00-17.00
Phz-1,6-di-COOH
I
K
16A1
16B1
23aP
RT: 6.71
6.57
6.47
6.28
6.58
6.45
6.32
6.18
6.39
6.45
Time (min)
16A1_10X_stepped #3338 RT: 6.50 AV: 1 NL: 5.67E5
T: FTMS + p ESI d Full ms2 269.0555@hcd53.33 [50.0000-295.4566]
II
MS/MS scan
269.0555
179.0602
251.0449
252.0513
270.0605
224.0577
180.0642
178.0521
206.0472
223.0502
Relative Abundance
m/z

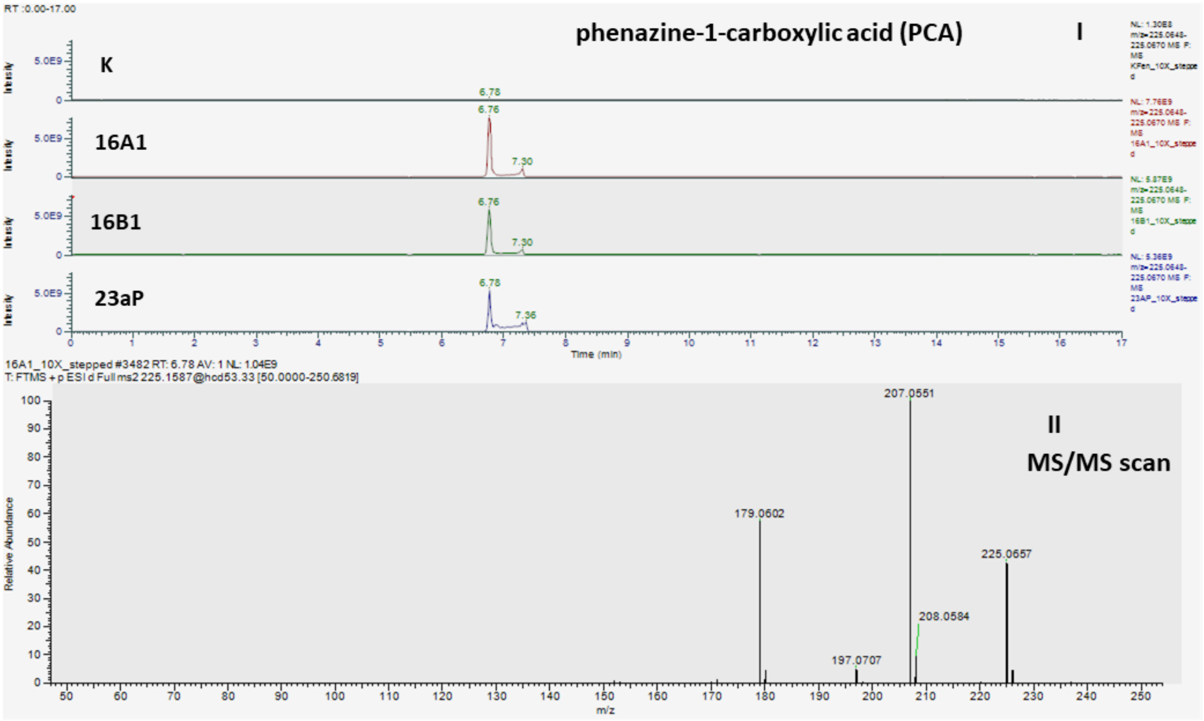
RT :0.00-17.00
phenazine-1-carboxylic acid (PCA)
I
K
16A1
16B1
23aP
6.78
6.76
7.30
6.76
7.30
6.78
7.36
Time (min)
16A1_10X_stepped #3482 RT: 6.78 AV: 1 NL: 1.04E9
T: FTMS + p ESI d Full ms2 225.1587@hcd53.33 [50.0000-250.6819]
II
MS/MS scan
207.0551
179.0602
225.0657
208.0584
197.0707
Relative Abundance
m/z

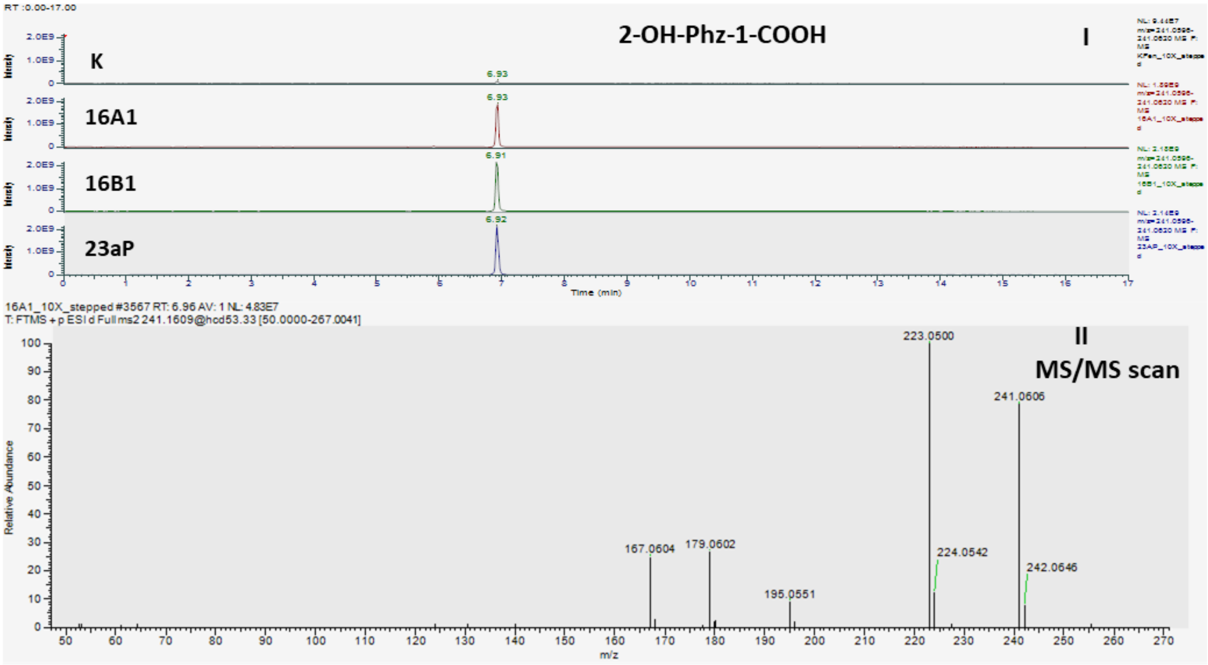
RT :0.00-17.00
2-OH-Phz-1-COOH
I
K
16A1
16B1
23aP
6.93
6.93
6.91
6.92
Time (min)
16A1_10X_stepped #3567 RT: 6.96 AV: 1 NL: 4.83E7
T: FTMS + p ESI d Full ms2 241.1609@hcd53.33 [50.0000-267.0041]
II
MS/MS scan
223.0500
241.0606
167.0604
179.0602
195.0551
224.0542
242.0646
Relative Abundance
m/z

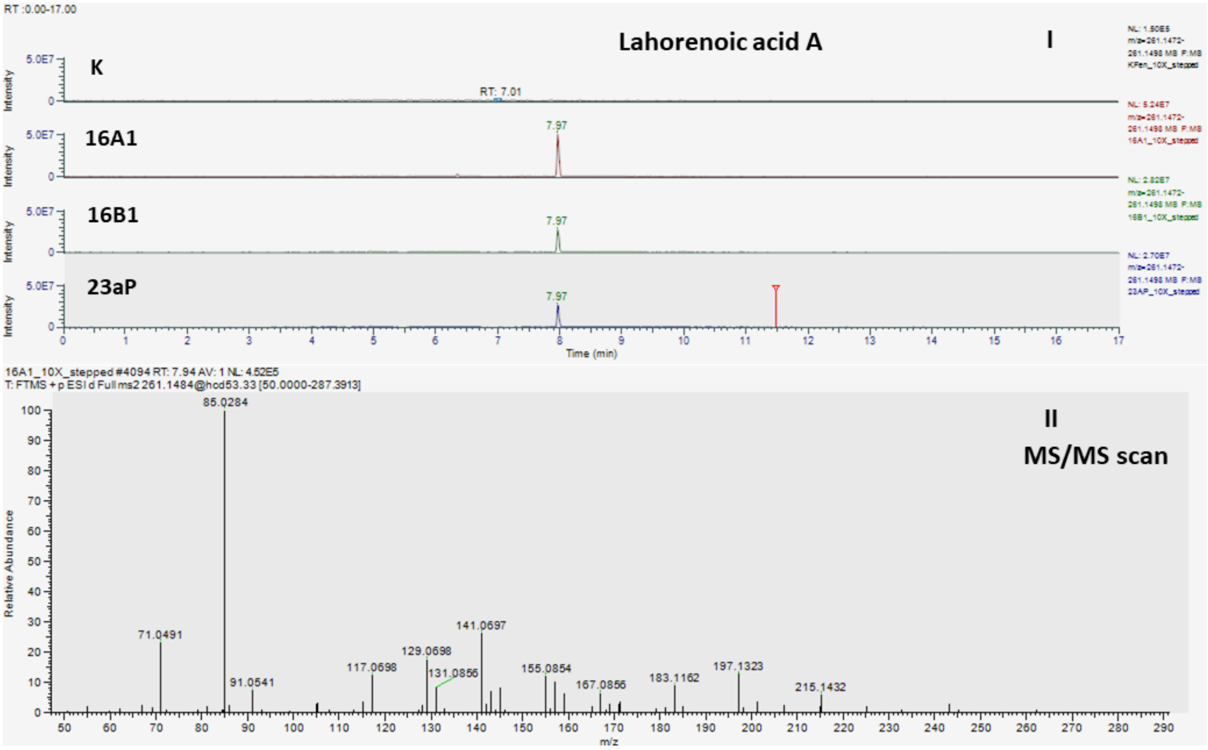
RT :0.00-17.00
Lahorenoic acid A
I
K
16A1
16B1
23aP
RT: 7.01
7.97
7.97
7.97
Intensity
Time (min)
16A1_10X_stepped #4094 RT: 7.94 AV: 1 NL: 4.52E5
T: FTMS + p ESI d Full ms2 261.1484@hcd53.33 [50.0000-287.3913]
II
MS/MS scan
85.0284
71.0491
91.0541
117.0698
129.0698
131.0856
141.0697
155.0854
167.0856
183.1162
197.1323
215.1432
Relative Abundance
m/z

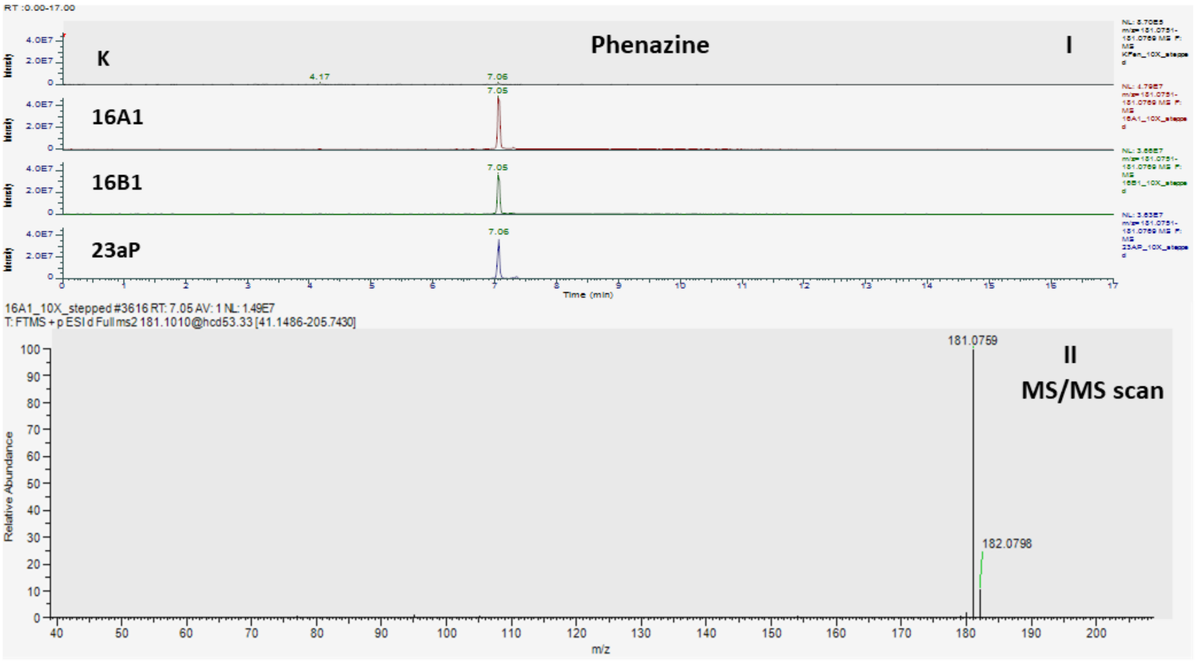
RT :0.00-17.00
Phenazine
I
K
16A1
16B1
23aP
4.17
7.06
7.05
7.05
7.06
Time (min)
16A1_10X_stepped #3616 RT: 7.05 AV: 1 NL: 1.49E7
T: FTMS + p ESI d Full ms2 181.1010@hcd53.33 [41.1486-205.7430]
181.0759
182.0798
II
MS/MS scan
Relative Abundance
m/z

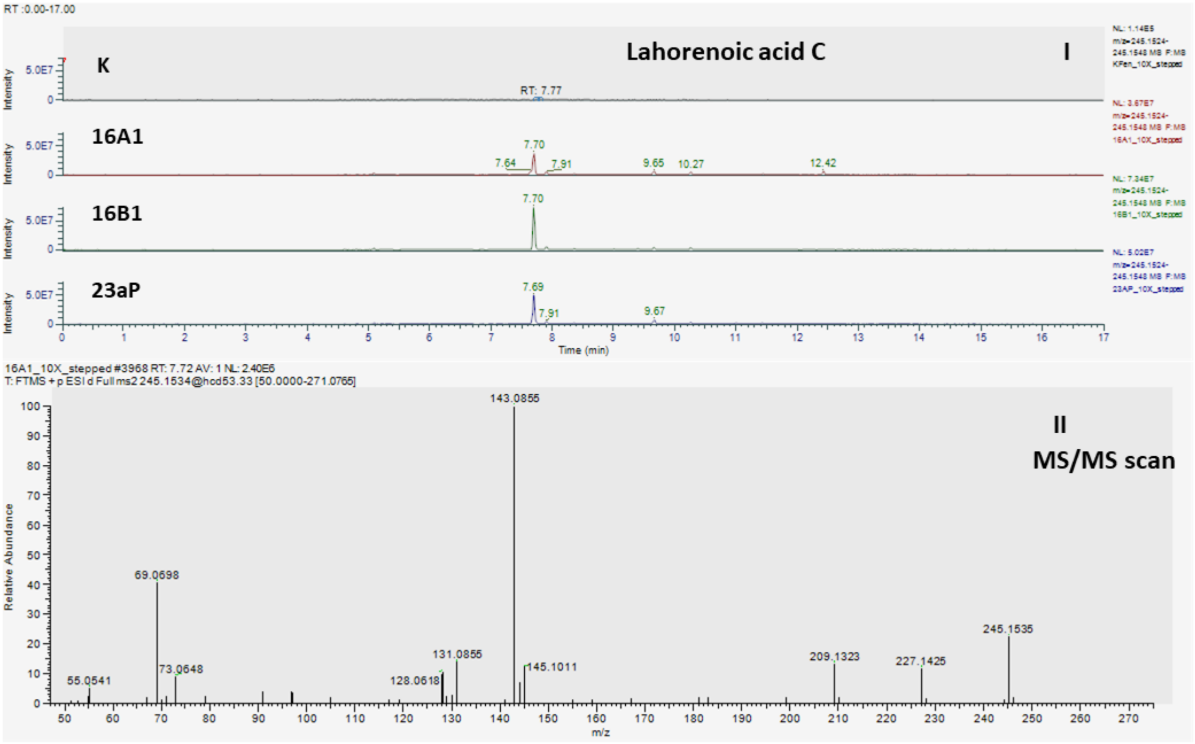
RT :0.00-17.00
Lahorenoic acid C
I
K
16A1
16B1
23aP
RT: 7.77
7.70
7.64
7.91
9.65
10.27
12.42
7.70
7.69
7.91
9.67
Intensity
Time (min)
16A1_10X_stepped #3968 RT: 7.72 AV: 1 NL: 2.40E6
T: FTMS + p ESI d Full ms2 245.1534@hcd53.33 [50.0000-271.0765]
143.0855
69.0698
55.0541
73.0648
128.0618
131.0855
145.1011
209.1323
227.1425
245.1535
II
MS/MS scan
Relative Abundance
m/z

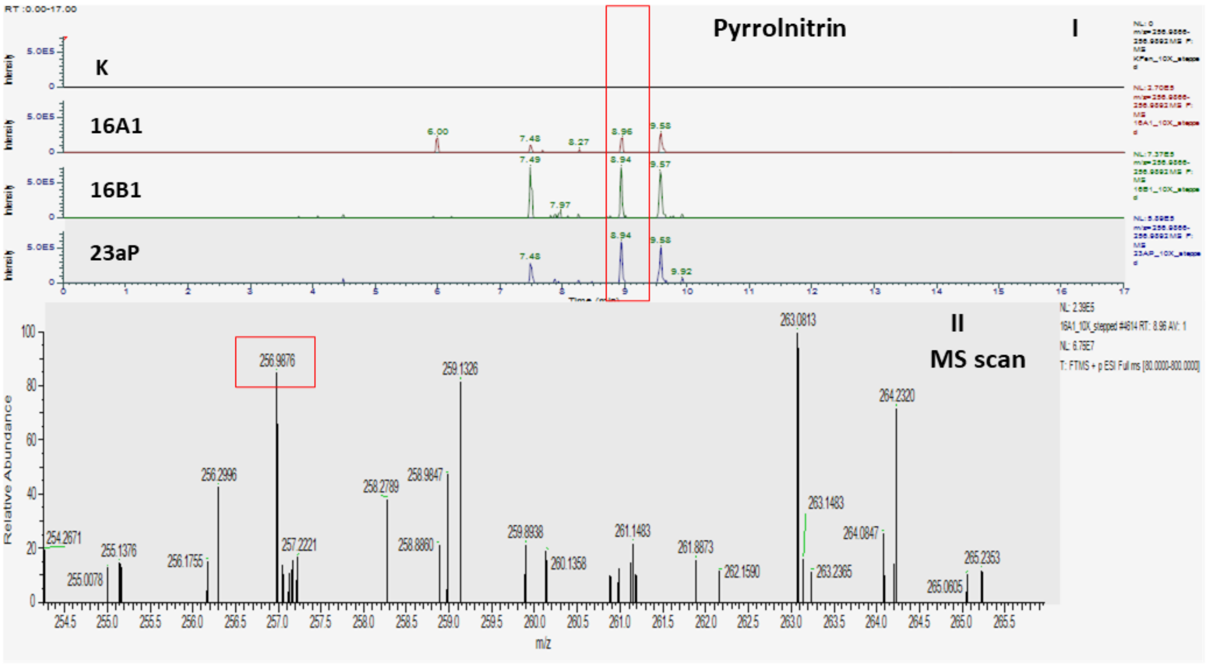

Figure S3. Secondary metabolites identified in cell-free supernatants of 16A1, 16B1, and 23aP *P. chlororaphis* cultures. (I) ionograms for particular compounds, and (II) MS or MS/MS scans registered for particular compounds in LC-MS or LC-MS/MS analysis used for their identification.

2.4. Molecular characteristics of some genes for antifungal antibiotics

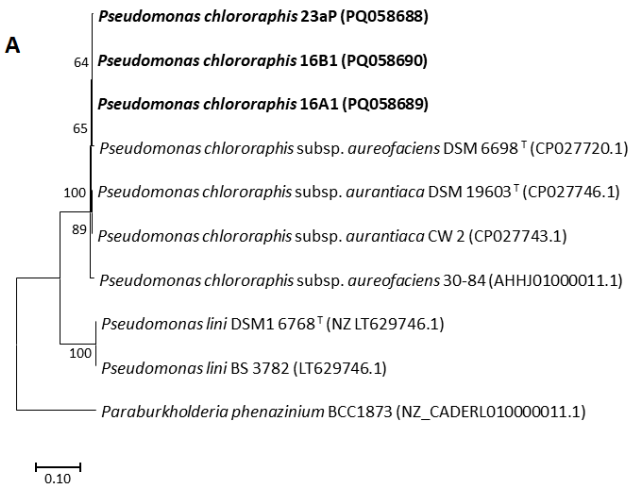

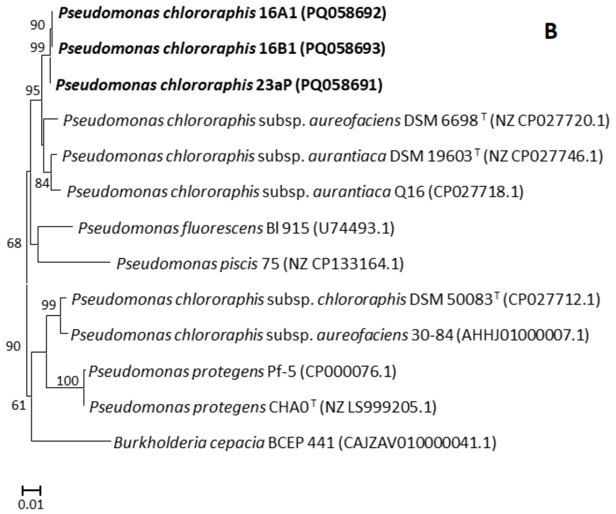

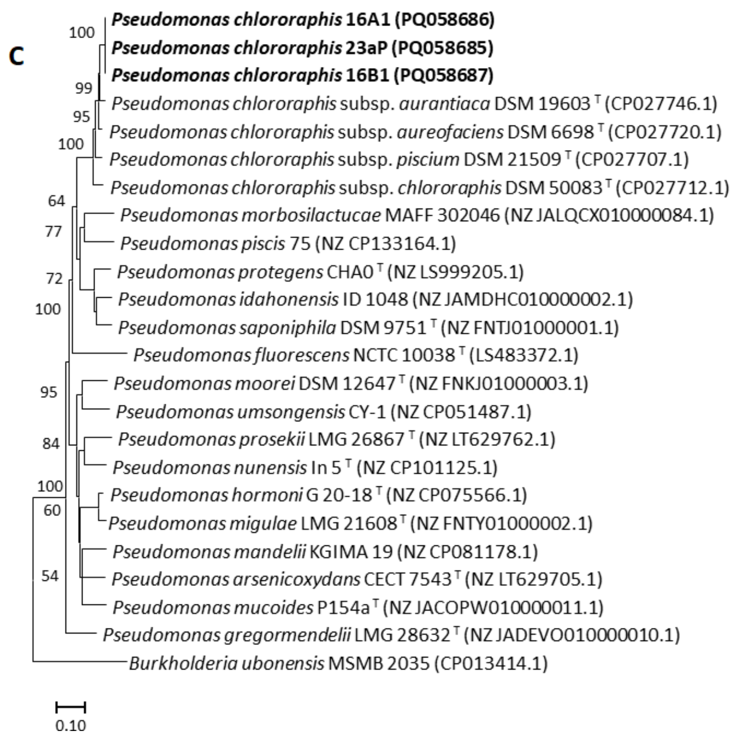

Figure S4. Maximum likelihood phylogenetic trees based on 701 bp fragments of the *phzO* gene (**A**), 694 bp fragments of the *prnC* gene (**B**), and 764 bp fragments of the *phzF* gene (**C**) constructed using the Neighbor-Joining (NJ) method [58]. Bootstrap values [%] higher than 50 are shown next to the branches. The scale bar represents 0.05 substitutions per nucleotide position.

2.4. Molecular characteristics of genes for antifungal antibiotics

Table S2. Primers used in this study

Primer	Sequence	Target	Properties	Size (bp)	Reference
**Phl2a**<br>**Phl2b**	**GAGGACGTCGAAGACCACCA**<br>**ACCGCAGCATCGTGTATGAG**	*phl*<br>**(2,4-diacetylphloroglucinol)**	**antifungal**	**745**	**[56]**
**2989_5′ENTR**<br>**2989_3′ ENTR**	**CACCTCTACAATCACCAGGGCG**<br>**TTCGCCGTCGATGGAACC**	*rzxB*<br>**(rhizoxin)**	**antifugal/**<br>**antibiotic**	**1394**	**[56]**
**Aca**<br>**Acb**	**CGGAGCATGGACCCCCAGC**<br>**ACGATGTGCTCGGCGTAC**	*hcnBC*<br>**(hydrogen cyanide)**	**antifungal**	**587**	**[56]**
**PltBf**<br>**PltBr**	**CGGAGCATGGACCCCCAGC**<br>**GTGCCCGATATTGGTCTTGACCGAG**	*plt*<br>**(pyoluteorin)**	**antifungal**	**792**	**[56]**
**prnBF**<br>**prnBR**	**GTGGAWCGCACCTTGRASCGG**<br>**TCAGGATTCRTCGAGCRCGGC**	*prnB*<br>**(pyrrolnitrin)**	**antifungal/ antibacterial**	**1080**	**This study**
**hcnF**<br>**hcnR**	**GGYGGCGGYGTGATCGGSGC**<br>**AACGGBGTGATRTCCAGCGGC**	*hcn*<br>**(hydrogen**	**antifungal/ antibacterial**	**1220**	**This study**

		cyanide)			
pltF<br>pltR	ATGAAAGCGACAGACACTAAA<br>GATGTGGTCGTTGTCCTTGCG	*plt*<br>(pyoluteorin)	antifungal/ antibacterial	888	This study
prnCF<br>prnCR	CCSGCCAGGAGCACGACCCGA<br>GATGAASCCGTTCGCGTGYAG	*prnC*<br>(pyrrolnitrin)	antifungal	694	This study
phzFF<br>phzFR	ATGCGCCGATTCGATTTCAA<br>CTACAACGTCAGGCTGCCCT	*phzF*<br>(phenazine)	antifungal/ antibacterial	843	This study
phzOF<br>phzOF	ATGCTAGATYTTCAAAACAA<br>CTATTTGGCGTTGAGCCCCA	*phzO*<br>(2-OH-PCA)	antifungal/ antibacterial	1476	This study
